# *Commiphora myrrha* resin extract inhibits the biofilms and quorum sensing controlled virulence factors of Gram-negative foodborne bacterial pathogens

**DOI:** 10.3389/fmicb.2025.1668863

**Published:** 2025-12-31

**Authors:** Nasser A. Al-Shabib, Fohad Mabood Husain, Faizan Abul Qais, Nasir A. Siddiqui, Iftekhar Hassan, Javed Masood Khan, Nayla Munawar, Rosina Khan, Mohd Adil

**Affiliations:** 1 Department of Food Science and Nutrition, College of Food and Agriculture Sciences, King Saud University, Riyadh, Saudi Arabia; 2 Department of Biochemistry, School of Chemical and Life Sciences, Jamia Hamdard, New Delhi, India; 3 Department of Pharmacognosy, College of Pharmacy, King Saud University, Riyadh, Saudi Arabia; 4 Department of Zoology, College of Science, King Saud University, Riyadh, Saudi Arabia; 5 Department of Chemistry, College of Science, United Arabs Emirates University, Al Ain, United Arab Emirates; 6 Department of Plant, Food and Environmental Sciences, Dalhousie University, Truro, NS, Canada

**Keywords:** *Commiphora myrrha*, biofilms, virulence, GC/MS, molecular docking, molecular simulation

## Abstract

Antimicrobial resistance (AMR) is a global health threat. Multi-drug-resistant pathogens now cause significant mortality worldwide. Widespread antibiotic misuse has fueled resistance, prompting interest in antivirulence approaches over traditional bactericidal drugs. Targeting biofilms and quorum sensing (QS) is a promising antimicrobial strategy, recognized as an effective inhibitor. In the current study, *Commiphora myrrha* was assessed on the virulent traits and biofilms of Gram-negative bacteria (*Pseudomonas aeruginosa* PAO1, *Serratia marcescens* MTCC 97, *Chromobacterium violaceum* ATCC 12472, and *Proteus mirabilis* MTCC 425). Methanolic extract of *C. myrrha* resin was prepared, and MIC was determined using the microdilution method. At sub-MICs, violacein production, QS-regulated virulence factors and biofilm development were estimated using spectroscopic assays. Phytochemicals were investigated using GC/MS analysis. Molecular docking was conducted between the QS-associated proteins (LasR, RhlR, and CviR) and the most abundant phytocompound of *C. myrrha*. MIC of CMRE against test strains was in the range of 0.5, and 2 mg/ml. CMRE reduced the violacein production in *C. violaceum* 12472 by 82.7%. In *P. aeruginosa* PAO1, production of virulence factors was reduced by >70%. The cell surface hydrophobicity was decreased to 18.9% compared to the control cells of *P. aeruginosa* PAO1 (76.4%). CMRE at ½ × MIC resulted in reduced biofilm formation in the range of 69.1–76.9%. A similar dose-dependent effect was observed on the exopolysaccharides production of the tested Gram-negative bacteria. Curzerene was identified as the most abundant (18.56%) phytoconstituent. Molecular docking revealed that curzerene interacted at the active sites of the tested proteins. Finally, molecular simulations validated the stability of curzerene with these proteins under an aqueous environment. The findings of this study may prove to be useful in the development of new anti-virulence bacterial drugs against Gram-negative bacteria.

## 1 Introduction

The global emergence and spread of antimicrobial resistance (AMR) pose a serious threat to human health. Infections caused by multidrug-resistant pathogens are now a leading cause of death worldwide (Murray et al., 2022). The severity of the AMR problem is underscored by the fact that methicillin-resistant *S. aureus* (MRSA) alone claims more lives than the combined death toll from emphysema, HIV/AIDS, Parkinson’s disease, and homicide (Llor and Bjerrum, 2014). The discovery of antibiotics in the early 20th century marked a significant milestone in human healthcare. However, pathogens have gradually developed resistance to these life-saving drugs. Improper antibiotic use, both underdosing and overdosing, has been a major driver of AMR (Byarugaba, 2004; Laxminarayan et al., 2013). Additionally, the widespread use of antibiotics in livestock and poultry has contributed to the emergence of resistant strains (Woolhouse et al., 2015). There are reports of patients prematurely discontinuing antibiotic treatment, only to later suffer from more severe infections caused by resistant variants (Okeke et al., 1999).

Over the past two decades, the discovery of novel antimicrobials has declined despite the rising burden of antimicrobial resistance (Ahmad et al., 2019). As a consequence of this stagnation, when microbes are exposed to higher-than-inhibitory doses of antibiotics, they experience selection pressure, leading to the acquisition of specific resistance mechanisms (Witte, 2000; Abushaheen et al., 2020). Given the rise in AMR and the low pace of antibiotic discovery have prompted to explore alternative interventions. One promising approach is to identify chemical compounds that do not directly inhibit bacterial growth but instead reduce pathogenicity (Ahmad et al., 2019). Such drugs could potentially minimize the likelihood of resistance development. Additionally, biofilms play a crucial role in microbial infections, and both biofilm formation and quorum sensing (QS) offer new avenues for antimicrobial drug discovery. Interestingly, plants may serve as a rich source of biofilm and QS inhibitors, given their ability to produce a diverse array of bioactive compounds.

*Pseudomonas aeruginosa* is a Gram-negative pathogen frequently implicated in hospital-acquired infections, most notably pneumonia and severe infections in immunocompromised individuals. It employs two primary acyl-homoserine-lactone circuits, the LasI/LasR and the RhlI/RhlR systems, which regulate key virulence traits including protease production, biofilm formation and toxin secretion (Sultan et al., 2021). In contrast, *Chromobacterium violaceum*, commonly found in soil and water, causes rare infections that can quickly spread to organs such as the liver, lungs, and spleen, often leading to severe sepsis with high mortality (Venkatramanan and Nalini, 2024). The bacterium is resistant to several antibiotics, especially some beta-lactams, but is typically sensitive to carbapenems and quinolones (Pei et al., 2024). It is known for producing the QS-regulated purple pigment violacein and forming robust biofilms. Furthermore, because its QS system closely resembles that of many other Gram-negative bacteria, *C. violaceum* serves as a useful model for testing both natural and synthetic QS inhibitors (Dimitrova et al., 2023). Similarly, the biofilm-associated resilience of *Serratia marcescens* and *Proteus mirabilis* poses significant challenges for clinical management and infection control (Armbruster et al., 2018; Zivkovic Zaric et al., 2023). Beyond clinical infections, these bacteria are found in food and food-processing contexts where persistent biofilms allow for surface colonization and cross-contamination, highlighting their importance to both public health and food safety (Moy and Sharma, 2017). This dual significance supports their inclusion in the current investigation.

Bacteria engage in coordinated behavior based on cell density, a phenomenon known as QS (Miller and Bassler, 2001). During QS, bacteria produce signal molecules called autoinducers (AIs). In Gram-negative bacteria, acylated homoserine lactones (AHLs) serve as the primary AIs (Fuqua et al., 2001). When the concentration of AIs reaches a critical level in the bacterial environment, it triggers the activation of a distinct set of genes that remain dormant at lower cell densities (Rutherford and Bassler, 2012). These AIs are recognized by receptors on the bacterial cell surface. Interestingly, many of the genes expressed during QS are secondary, and they are not essential for normal bacterial growth and division. These secondary genes may include factors related to virulence and drug resistance.

*Commiphora myrrh*, a flowering plant classified within the Burseraceae family, is commonly found in the southern part of Arabia, north-eastern Africa, Somalia, and Kenya (Alyafei, 2020). Traditionally, its resins have been used to treat a variety of conditions, including mouth ulcers, wounds, fractures, aches, microbial infections, stomach disorders, and inflammatory diseases (Batiha et al., 2023). In Unani medicine, myrrh gums serve as astringents, antiseptics, carminatives, anthelmintics, expectorants, emmenagogues, and stomachics (Batiha et al., 2023). It’s essential oil is widely used in cosmetics, aromatherapy, and the fragrance industry. Notably, in rat studies, myrrh hydro suspension demonstrated gastric mucosal protection against various ulcer-inducing agents, while the ethanolic extract exhibited anti-inflammatory effects in mice, both in acute and chronic inflammation scenarios (Akbar, 2020). Ethnobotanical evidence indicates that *C. myrrha* resin has long been applied to infected wounds, oral lesions, and skin inflammations to prevent suppuration and promote tissue repair (Eid, 2021; Suliman et al., 2022). Moreover, *C. myrrha* or its essential oil has shown antibacterial activity in several *in-vitro* studies against Gram-positive and negative pathogens, further supporting its traditional use in infection management (AL-Abdalall, 2016; Mustafa et al., 2022; Izzeldien et al., 2020; Khalil et al., 2020). These evidences substantiate the rationale for assessing the anti-virulence and anti-biofilm properties of *C. myrrha*. Reports on the broad-spectrum anti-infective and antibiofilm efficacy of C. *myrrha* are scarce. In the present work, we have examined the effect of *C. myrrha* resin extract (CMRE) in the biofilms and QS-regulated virulent traits of Gram-negative bacteria. We also performed GC/MS analysis to dissect the major phytocompounds present in the extract. Lastly, molecular docking and molecular dynamics simulations were carried out to explore the possible mechanistic insights.

## 2 Materials and methods

### 2.1 Collection and plant material and preparation of extract

*C. myrrha* resins was procured from commercial herbal supplier (CLENZ^®^, Riyadh, Saudi Arabia; 100% natural myrrh resin, 50 g pack). The material was cleaned to remove surface impurities. The resins were air-dried at room temperature and ground into fine powder. Briefly, 100 g of powdered resin was extracted in 500 mL of methanol (Sigma-Aldrich) for 5 days with intermittent shaking at room temperature, protected from direct light. The mixture was then centrifuged at 10,000 rpm for 10 min and filtered through Whatman No. 1 filter paper (Whatman Ltd., England). The filtrate was concentrated using a rotary evaporator () at 40°C. The concentrated *C. myrrha* resin extract (CMRE) was stored at 4°C until further use.

### 2.2 Bacterial strains used in this study and their growth conditions

For this study, we assessed the impact of CMRE on four Gram-negative bacteria, namely *Pseudomonas aeruginosa* PAO1, *Serratia marcescens* MTCC 97, *Chromobacterium violaceum* 12472, and *Proteus mirabilis* MTCC 425. Each strain was grown in Luria-Bertani (LB) broth, adhering to its specific growth requirements. Unless mentioned otherwise, all the assays related to biofilm and QS were performed in LB broth.

### 2.3 Examination of minimum inhibitory concentration

The MIC of *C. myrrah* resin extract (CMRE) against test Gram-negative pathogens was determined using TTC dye, as described previously (Maheshwari et al., 2019). Briefly, CMRE was serially diluted in LB broth within a 96-well microtiter plate to obtain a range of concentrations. Hundred μl of respective bacterial culture (5 × 10^5^ CFU/mL) were added to each well. Plates were incubated at 37°C overnight, after which 20 μL of TTC solution (2 mg/mL) was added to each well and incubated for 20 min in the dark. Wells developing a pink/red color were considered to contain viable cells, whereas the absence of color indicated growth inhibition. To confirm bacteriostatic or bactericidal effects, wells showing no color change were streaked onto LB agar and incubated. The MIC was defined as the lowest concentration of CMRE that prevented visible growth. All biofilm and QS assays were performed at sub-inhibitory concentrations (sub-MICs), i.e., ½ × MIC, ¼ × MIC, ⅛ × MIC, and ¹⁄₁₆ × MIC.

### 2.4 Assessment of violacein pigment in *C. violaceum* 12472

The process for extracting and quantifying violacein was adopted from an earlier method (Matz et al., 2004). In summary, *C. violaceum* 12472 was grown overnight in LB medium, with and without sub-MICs of CMRE. Groups without any treatment were taken as control. After incubation, the cultures were centrifuged to collect the cells. The resulting cell pellet was resuspended in DMSO (1 mL) and vigorously vortexed for dissolution of violacein pigment. The mixture was then centrifuged again to remove the debris and remaining cells. The absorbance of the supernatant was measured at 585 nm to determine the violacein concentration.

### 2.5 Assessment of virulence factors in *P. aeruginosa* PAO1

To enhance pyocyanin production, the assay was conducted using *Pseudomonas* broth (PB) medium, which contains 20 g/L peptone, 1.4 g/L MgCl_2_, and 10 g/L K_2_SO_4_, as previously described. *P. aeruginosa* PAO1 was grown in PB medium both without and with sub-MICs of CMRE for 18 h. After centrifugation, the culture supernatant was collected. A 5 mL culture supernatant was extracted with 3 mL of chloroform, and the aqueous phase was discarded. Next, the organic phase was subsequently re-extracted into 1.2 mL of 0.2 N HCl. Finally, the absorbance of the resulting pink/deep red aqueous phase was measured at 520 nm. The pyocyanin concentration, expressed in μg/mL, was calculated by multiplying the OD520 by 17.072 (Kurachi, 1958).

The pyoverdin assay was conducted following the previously described method (Ankenbauer et al., 1985). In summary, *P. aeruginosa* PAO1 cultures, grown overnight with and without sub-MICs of CMRE, were centrifuged to obtain a supernatant. A 100 μL aliquot of this supernatant was combined with 900 μL of 50 mM Tris-HCl buffer (pH 7.4). The fluorescence of the mixture was then measured at an emission wavelength of 460 nm, with excitation at 400 nm, using a spectrofluorometer.

The rhamnolipid assay was conducted using a modified orcinol method as previously described (Husain et al., 2017). A 300 μL aliquot of culture supernatant from both CMRE-treated and untreated *P. aeruginosa* PAO1 cultures was extracted with 600 μL of diethyl ether. The organic phase was then separated and evaporated to dryness at 37°C, after which it was reconstituted in 100 μL of deionized water. To each sample, 900 μL of orcinol solution (0.19% orcinol in 53% H_2_SO_4_) was added. The reaction mixture was heated at 80°C for 30 min, then allowed to cool to room temperature for 15 min. The absorbance of the resulting solution was measured at 421 nm.

Cell surface hydrophobicity (CSH) was assessed using xylene, based on a previously described method (Rosenberg et al., 1980). Briefly, 1 ml of an overnight culture of *P. aeruginosa* PAO1 was mixed with 0.1 ml of xylene and sub-MICs of CMRE. The mixture was vigorously vortexed and then allowed to sit for 10 min at room temperature to enable phase separation. The absorbance of the aqueous phase was measured at 530 nm. The percentage of hydrophobicity was then calculated using the appropriate formula.

$$\%hydrophobicity=\left[1-\frac{O\,D\,a\,f\,t\,e\,r\,v\,o\,r\,t\,e\,x\,i\,n\,g}{O\,D\,b\,e\,f\,o\,r\,e\,v\,o\,r\,t\,e\,x\,i\,n\,g}\right]\times 100\;\left(1\right)$$

To assess swimming motility, 5 μL of an overnight culture of *P. aeruginosa* PAO1 was placed on LB agar plates (0.3% agar) and allowed to dry at room temperature. Plates without CMRE served as controls. The plates were then incubated for 18 h, and the swimming motility was evaluated by measuring the diameter of the swarm zone. In the above QS assays cells without any treatment were taken as control.

### 2.6 Assessment of biofilms of Gram-negative bacteria

Biofilm inhibition studies were performed using a 96-well culture plate, following previously established protocols (Husain et al., 2017). Overnight cultures of test bacteria were added to the wells, each containing 0.15 mL of LB medium. Different sub-MICs of CMRE were used for the treatment groups, while control groups received no treatment. Antibiotic azithromycin was used as positive control for biofilm inhibition. The plate was incubated for 24 h. After incubation, planktonic cells and excess broth were removed by washing the wells with phosphate buffer. The plate was then air-dried for 15 min. Biofilms were stained with 0.2 mL of crystal violet for 20 min, followed by gentle washing. The bound dye was dissolved in ethanol, and the optical density was measured at 620 nm using a microplate reader.

### 2.7 Assessment of exopolysaccharides secreted by Gram-negative bacteria

The levels of exopolysaccharides in both untreated and CMRE-treated cultures were quantified using a standard procedure (Husain et al., 2018). In brief, the test bacteria were cultured with various sub-MICs of CMRE for 24 h, while the control group received no treatment. After incubation, the cultures were centrifuged to obtain cell-free supernatant. Chilled ethanol was then added to the supernatant in a 3:1 ratio, and the mixture was incubated overnight at 4°C to precipitate the EPS. The EPS levels were quantified using the Dubois method to estimate the sugar content (DuBois et al., 1956).

### 2.8 Gas chromatography/mass spectrometric analysis of CMRE

The CMRE underwent GC/MS analysis to identify its phytoconstituents. GC–MS analysis was performed using a Perkin Elmer Autosystem XL gas chromatograph coupled to a Turbomass mass spectrometer equipped with an electron ionization (EI) source, fitted with a PE-Wax capillary column (30 m × 0.25 mm., film thickness 0.25 μm). Helium served as the carrier gas, with the detector and injector temperatures set at 280 and 250°C, respectively. The initial oven temperature was set to rise from 100 to 250°C at a rate of 10°C per minute, with a 3-min hold at 250°C. The final oven temperature was increased from 250 to 280°C at a rate of 30°C per minute, with a 2-min hold at 280°C. Methanol was used for all dilutions. To identify the compounds, the mass spectra of the peaks obtained were compared with those in the NIST library, applying a minimum match probability threshold of ≥ 90% to ensure reliable identification. The compounds were named based on their percent peak area and retention time.

### 2.9 Molecular docking of curzerene with target proteins involved in quorum sensing

The anti-quorum-sensing sensing-potential of curzerene, identified through GC/MS analysis of CMRE, was further studied using molecular docking. The 3D structure of curzerene (CID: 12305301) was obtained from PubChem. The molecule was then converted to pdb format using Chimera 1.14. To achieve optimal binding conformations, the ligand was made flexible using MGL Tools-1.5.6, and the coordinate files were saved in pdbqt format. AutoDock Vina was employed to perform the docking studies (Trott and Olson, 2009). AutoDock Vina is known for its higher accuracy and faster performance compared to AutoDock4 (Qais et al., 2016). The 3D crystal structures of the receptor proteins (LasR, RhlR, and CviR) were downloaded from the Protein Data Bank. Water molecules and other non-protein atoms were removed. The non-polar hydrogen atoms were merged, and Kollman charges were added using MGL Tools-1.5.6 (Qais et al., 2017). The grid size was made in such a way as to cover the entire receptor molecule with a grid spacing set to 1 Å. The coordinates were saved in pdbqt format. The post-docking analysis was conducted using PyMOL 3.0.0, LigPlot^+^, and Discovery Studio 2024.

### 2.10 Molecular dynamics simulation

The stability and dynamics of curzerene with three proteins (LasR, RhlR, and CviR) were further explored using molecular simulations. These simulations were conducted with Gromacs 2018.1, utilizing the amber99sb-ILDN force field (Berendsen et al., 1995; Hornak et al., 2006). Protein’s topologies were generated using the pdb2gmx command in Gromacs, while the topology for curzerene was created using the AM1-BCC charge model in the Antechamber package of AmberTools24 (Sousa da Silva and Vranken, 2012). The topologies of proteins and curzerene were then combined manually. The complexes were solvated with TIP3P water model in cuboidal boxes, neutralized, and then added with 150 mM NaCl to mimic physiological conditions. A 1.0 nm spacing was maintained between the structures and the box edges to satisfy periodic boundary conditions (PBC). Energy minimization was performed to eliminate weak van der Waals contacts, with a maximum of 50,000 steps using the steepest descent method. The first equilibration phase was conducted at a constant temperature (310 K) and volume (NVT ensemble) using a V-rescale thermostat for 1,000 ps (Bussi et al., 2007). This was followed by a second equilibration phase at constant pressure and temperature (310 K) for another 1000 ps using a Parrinello-Rahman barostat, termed as VPT ensemble (Parrinello and Rahman, 1981). Both equilibration phases employed a short-range van der Waals cutoff of 1.2 nm. Subsequently, the systems underwent a 100 ns molecular simulation, with 5,000 frames saved from each trajectory. For the analysis of MD trajectories, the PBC correction was done to raw trajectories and only the coordinates of proteins/complexes were taken as out for further analysis. The calculation of RMSD, RMSF, Rg, and SASA were done using the *gmx rms*, *gmx rmsf*, *gmx gyrate*, and *gmx sasa* utilities in Gromacs 2018.1. The interaction energies between curzerene and the test proteins were calculated using MM-PBSA method (Kumari et al., 2014). For MM-PBSA calculation, 100 snapshots were extracted at 0.5 ns intervals from 50 to 100 ns of the trajectory.

### 2.11 Statistical analysis

The *in vitro* experiments performed in three replicates. The data is presented as average with standard deviation of triplicates. One-way ANOVA was used for statistical analysis to identify differences between treatment group followed by and Duncan’s Multiple Range Test (DMRT) for *post-hoc* comparison. Statistical significance was defined as a significance level of *p* < 0.05. Statistically significant differences between groups are shown by different superscript letters above the bars.

## 3 Results and discussion

### 3.1 Determination of MIC and selection of sub-MICs

TTC dye was employed for the determination of the MIC of CMRE against test bacterial pathogens. The MIC of CMRE against *P*. *aeruginosa PAO1*, *S*. *marcescens* MTCC 97, *C*. *violaceum* 12472, and *P*. *mirabilis* 425 were found to be 2, 1, 0.5, and 2 mg/mL, respectively. For the assessment of biofilms and virulence factors, only sub-MICs were chosen at ½ × MIC, ¼ × MIC, ⅛ × MIC, and ¹⁄₁₆ × MIC.

### 3.2 CMRE inhibits the violacein pigment in *C. violaceum* 12472

**FIGURE 1:**
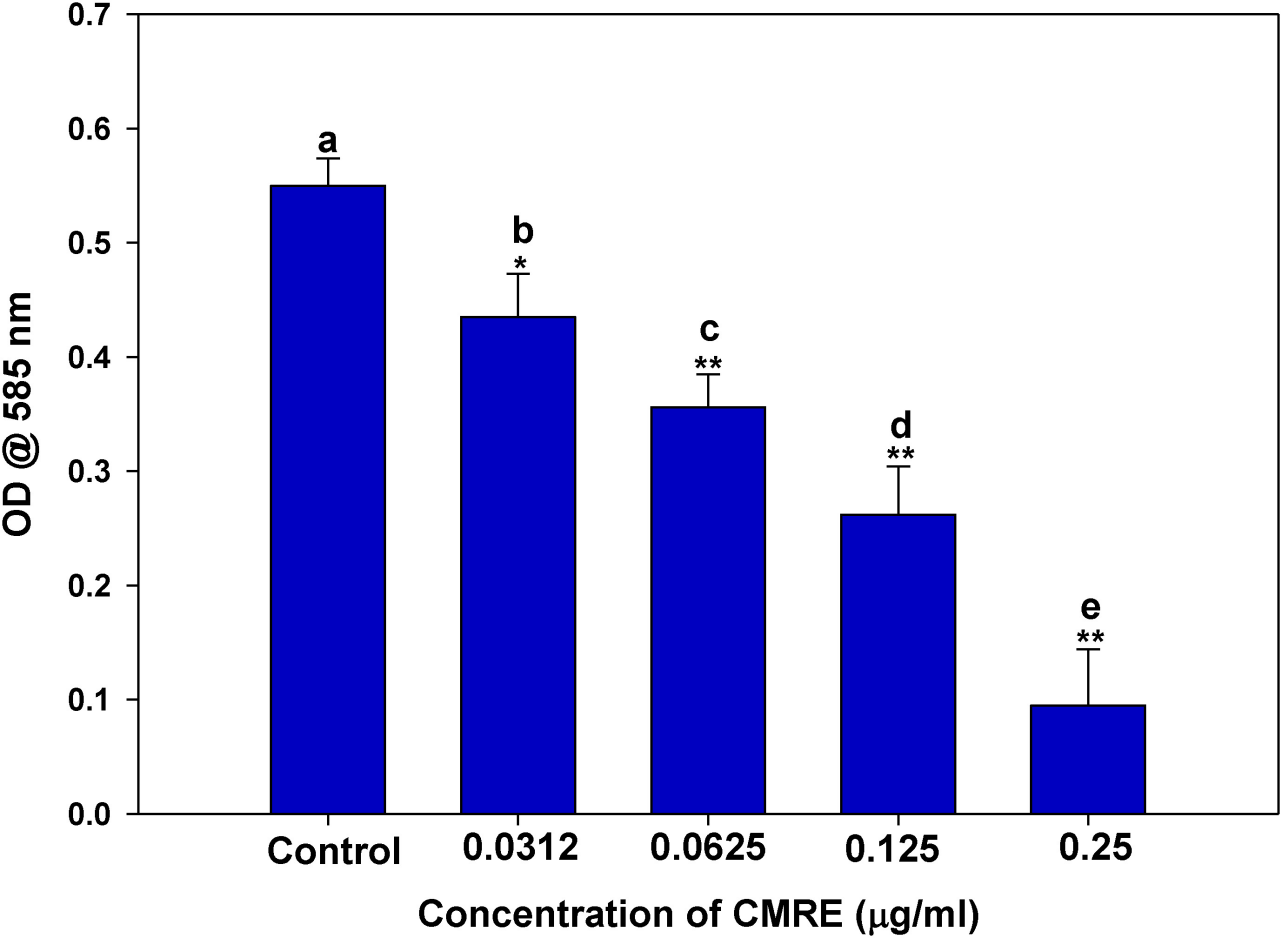
Quantitative determination of violacein inhibition in *C. violaceum* 12472 by sub-MICs of CMRE. Data is presented as mean of triplicates and bar denotes SD. Different letters above the bars indicate statistically significant differences among treatments (*p* < 0.05). * depicts *p* ≤ 0.05 wrt to control and ** depicts *p* ≤ 0.01 wrt to control. Means denoted by the different letters within parameter are significantly different at *p*≤0.05.

This spectrophotometric analysis aimed to measure the inhibition of violacein production by measuring the absorbance of violacein pigment at 585 nm. In the control group, the absorbance of violacein pigment was 0.550; the absorbance decreased dose-dependently when treated with increasing sub-MICs of CMRE (Figure 1). When *C. violaceum* 12472 was exposed to ¹⁄₁₆ × MIC, ⅛ × MIC, and ¼ × MIC CMRE, there were reductions in pigment production, amounting to 20.9, 35.2, and 52.3%, respectively. The highest sub-MIC tested (½ × MIC) resulted in a substantial 82.7% inhibition of pigment production. These results strongly indicate that CMRE has a notable inhibitory effect on the QS-regulated production of violacein in *C. violaceum* 12472. In this bacterium, violacein production is regulated by the CviR system, and the observed reduction in pigment production suggests that the extract has potential as a QS inhibitor. Overall, CMRE demonstrated a significant capacity to inhibit violacein production, warranting further investigation into its mechanism of action. Various reports have emerged in the recent past that have demonstrated the role of plant extracts in impairing QS-regulated violacein production in *C. violaceum*. Ethanolic extract of *Passiflora edulis* was evidenced to reduce violacein production remarkably in a dose-dependent manner. It was inferred that the extract interferes with the QS system by inhibiting the production of acyl homoserine lactone (AHL) (Venkatramanan et al., 2020).

### 3.3 Inhibition of QS-controlled virulence factors in *P. aeruginosa* PAO1 by CMRE

**FIGURE 2:**
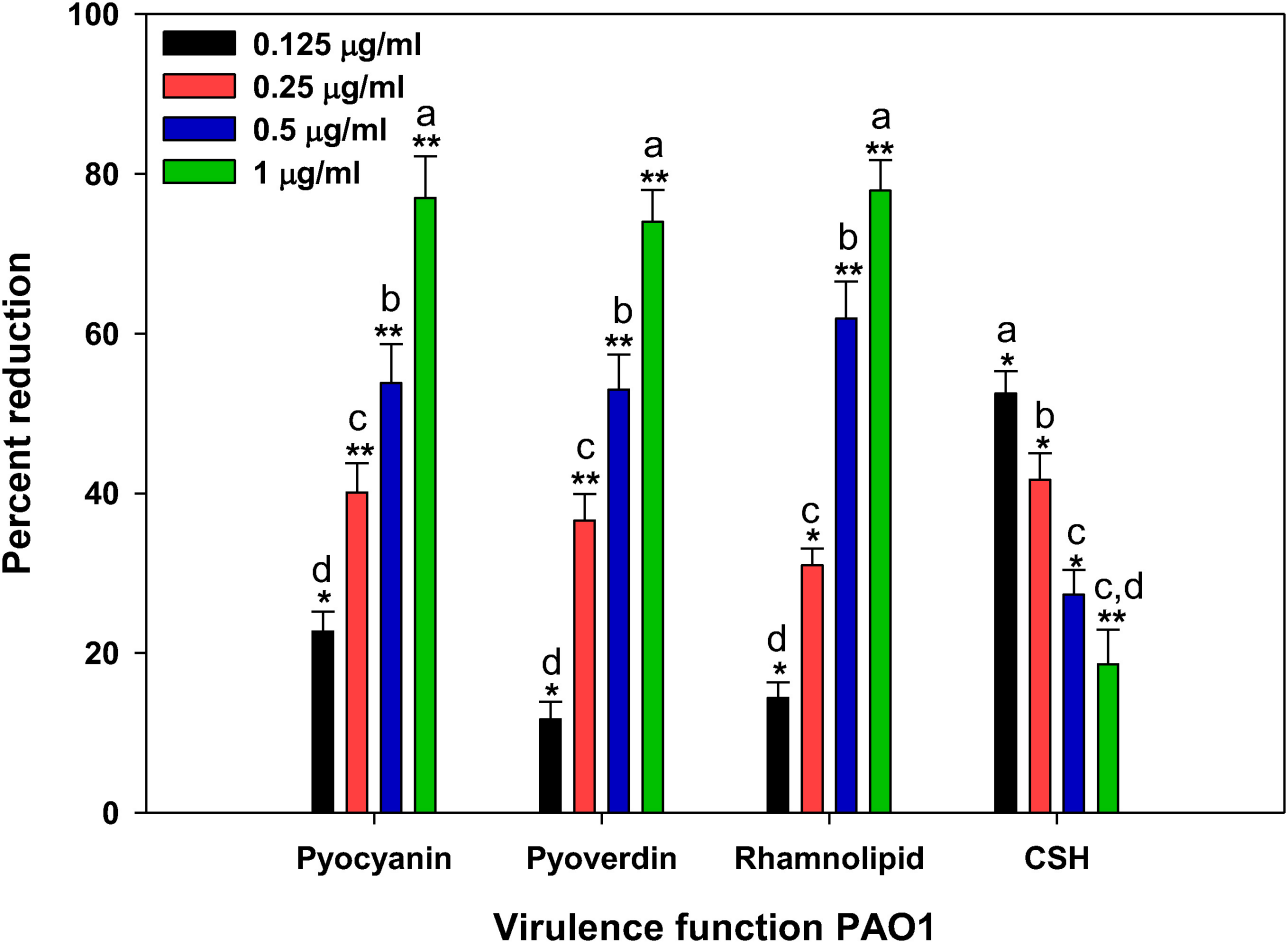
Effect of CMRE on virulence functions of *P. aeruginosa* PAO1. Data is presented as mean of triplicates and bar denotes SD. Different letters above the bars indicate statistically significant differences among treatments (*p* < 0.05). * depicts *p* ≤ 0.05 wrt to control and ** depicts *p* ≤ 0.01 wrt to control. Means denoted by the different letters within parameter are significantly different at *p*≤0.05.

Pyocyanin, a pigment known for its distinctive blue-green color, serves as a virulence factor in *P. aeruginosa* infections. We have found that treatment with *CMRE* resulted in a notable reduction in pyocyanin production, as illustrated in Figure 2. Specifically, ¹⁄₁₆ × MIC and ⅛ × MIC, and ¼ × MIC of CMRE led to reductions of 22.7, 40.1 and 53.8% in pyocyanin levels, respectively. At ½ × MIC, more than 75% inhibition of this tested pigment was found. This pigment plays a critical role in the pathogenicity of *P. aeruginosa* by disrupting various cellular processes (Ran et al., 2003). Additionally, the severity of the disease is closely linked to the oxidative stress induced by pyocyanin (Hunter et al., 2012). Notably, pyocyanin also undermines the host’s defense mechanisms, contributing to increased apoptosis in human neutrophils and promoting biofilm formation (Das et al., 2015). The observed inhibitory effect of CMRE on pyocyanin production suggests a promising strategy for mitigating *P. aeruginosa* virulence. By reducing pyocyanin levels, CMRE could potentially contribute to attenuating the pathogenic impact of *P. aeruginosa*. These findings exhibit the therapeutic promise of CMRE in combating *P. aeruginosa* infections.

Pyoverdin, a fluorescent siderophore secreted by *P. aeruginosa*, significantly contributes to the bacterium’s virulence and ability to infect hosts (Peek et al., 2012). In our study, we observed that CMRE (at ¹⁄₁₆ × MIC and ⅛ × MIC) reduced pyoverdin secretion by 11.7 and 36.6%, respectively, compared to the control *P. aeruginosa* PAO1, as shown in Figure 2. At higher doses at ¼ × MIC and ½ × MIC, the inhibition of pyoverdin was found as 53 and 74%, respectively. Pyoverdin plays a critical role in *P. aeruginosa* pathogenesis by binding to transferrin protein, resulting in iron deficiency within mammalian host tissues (Das et al., 2016). Furthermore, pyoverdin cleverly evades detection by lipocalin (associated with neutrophil gelatinase), allowing *P. aeruginosa* to thrive, especially in the lungs of cystic fibrosis patients (Peek et al., 2012). Consequently, any therapeutic agent that curtails pyoverdin production could potentially weaken the bacteria’s pathogenicity. A similar dose-dependent reduction in pyoverdine has been reported with other plant-derived QS or anti-virulence agents, indicating that suppression of siderophore synthesis is a recognized strategy for weakening *P. aeruginosa* pathogenicity (Rather et al., 2021a; Samreen and Ahmad, 2025).

The observed decrease in pyoverdin secretion in the presence of CMRE suggests a promising strategy for combating *P. aeruginosa* virulence. This finding holds significant implications for developing targeted approaches against *P. aeruginosa* infections, particularly in contexts like cystic fibrosis. Moreover, the application of CMRE to the bacterium’s growth revealed a suppressive effect on rhamnolipid production in *P. aeruginosa* PAO1, as illustrated in Figure 2. When tested at sub-MICs of ¹⁄₁₆ × MIC, ⅛ × MIC, ¼ × MIC, and ½ × MIC, CMRE led to reductions of 14.4, 31, and 61.9%, respectively, in rhamnolipid levels. Rhamnolipids, well-established for their role in QS-mediated motility (particularly swimming) and biofilm dispersal at infection sites, play a crucial role in *P. aeruginosa*’s pathogenicity (Köhler et al., 2000). These surfactant molecules contribute to surface motility and are indispensable for initiating biofilm formation (O’May and Tufenkji, 2011). The observed decline in rhamnolipid production in response to CMRE treatment is comparable to other studies highlighting the reduction in rhamnolipid by other plant extracts (Qais et al., 2021; Khan et al., 2024). Given that rhamnolipids are integral to bacterial motility and biofilm formation—both critical aspects of *P. aeruginosa*’s pathogenicity—the inhibitory effect exerted by CMRE shows its potential for disrupting bacterial virulence and mitigating biofilm-related infections.

We further examined the influence of CMRE on cell surface hydrophobicity (CSH), a crucial factor governing bacterial attachment and biofilm formation on solid surfaces (Salini and Pandian, 2015). Remarkably, CMRE led to a significant reduction in CSH. When exposed to ¹⁄₁₆ × MIC, ⅛ × MIC, ¼ × MIC, and ½ × MIC the CSH decreased to 18.7, 27.3, 41.7, and 52.5%, respectively (Figure 2). The finding corresponds to our earlier studies showing that green tea extract, a plant extract of ethyl acetate, efficiently lowers CSH and hinders biofilm formation in *S. marcescens* (Qais et al., 2019). This finding also aligns with prior research demonstrating that *A. graveolens* extract effectively reduces CSH and inhibits biofilm development in *S. marcescens* (Salini and Pandian, 2015). Moreover, in another study conducted by Bessalah et al. (2023) on *Escherichia coli* F17, eight tested plant extracts decreased cell-surface hydrophobicity to values between 20.3 and 45% (Bessalah et al., 2023). The changes in CSH are mainly caused by plant polyphenols and flavonoids, which interact with outer membrane proteins and lipopolysaccharides (LPS), leading to changes in surface charge and therefore reduced hydrophobicity (Liu et al., 2020). These results emphasize CMRE’s potential to modulate rhamnolipid production, a critical aspect with direct implications for bacterial pathogenesis and biofilm-related infections.

### 3.4 Effect of CMRE on the biofilm development of Gram-negative bacteria

**FIGURE 3:**
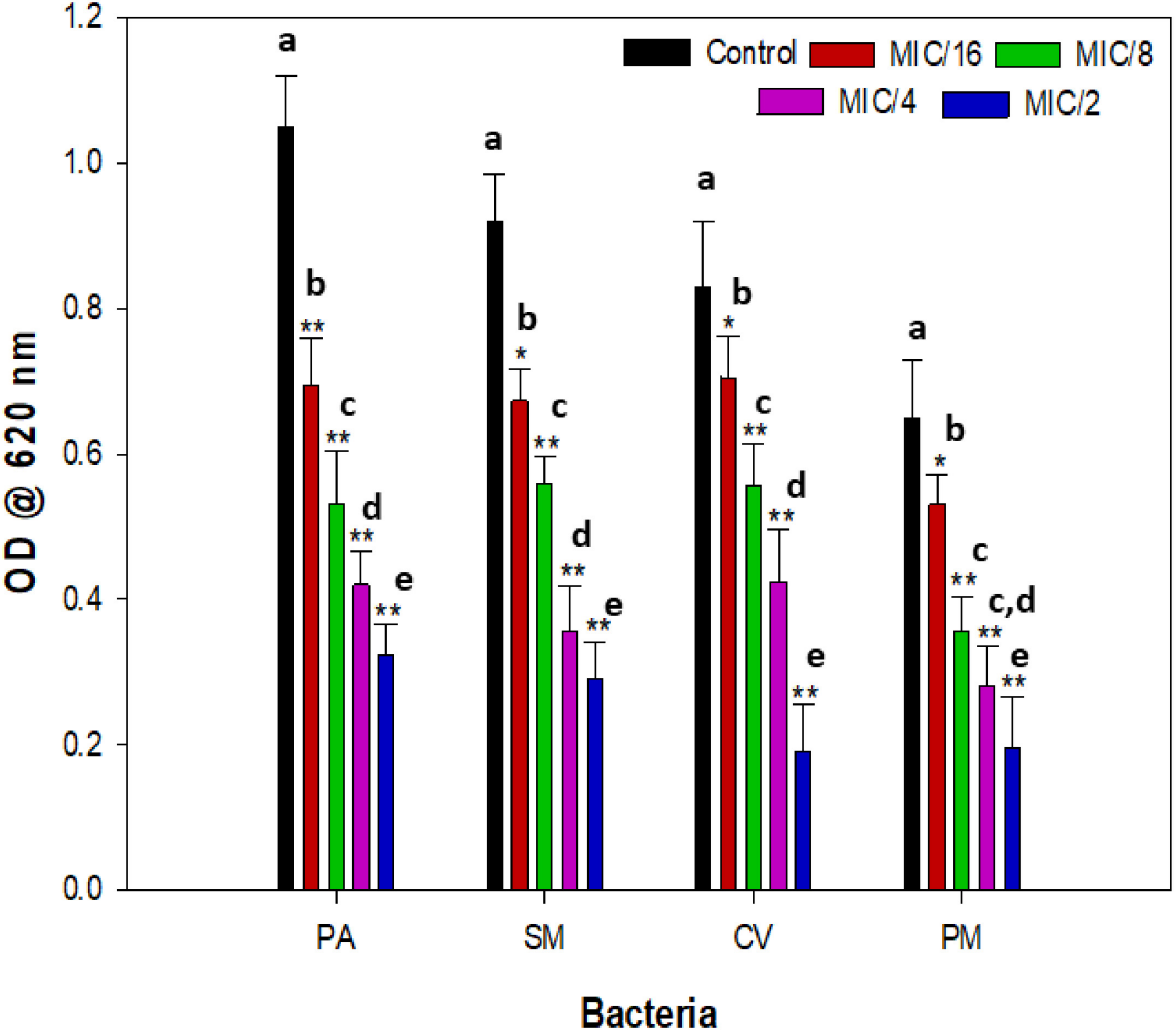
Biofilm inhibitory effect of sub-MICs of CMRE in test bacterial pathogens. Data is presented as mean of triplicates and bar denotes SD. Different letters above the bars indicate statistically significant differences among treatments (*p* < 0.05). * depicts *p* ≤ 0.05 wrt to control and ** depicts *p* ≤ 0.01 wrt to control. Means denoted by the different letters within parameter are significantly different at *p*≤0.05.

The pathogenicity of bacterial infections is linked to biofilms, which confer enhanced resistance to both chemical and physical treatments (Pompilio et al., 2015). Biofilm development is a highly organized and tightly regulated process; the process is linked with bacterial cellular communication, i.e., QS mechanisms (Qin et al., 2015). Remarkably, nearly 80% of human infections involve biofilms. The persistence of biofilms is also associated with the biofilm matrix, mainly composed of exopolysaccharides (Flemming and Wingender, 2010). In our prior research, we explored the anti-QS and antibiofilm activities of numerous plant extracts (Qais et al., 2021, 2024). Biofilms pose significant health concerns for their ability to confer drug resistance, resilience against host defense systems, and ability to withstand various stresses (de la Fuente-Núñez et al., 2013). Consequently, they contribute to the global prevalence of chronic bacterial infections (Subhadra et al., 2018; Sharma et al., 2019). A bacterial biofilm represents a complex structure—an organized aggregation of diverse bacterial colonies firmly attached to surfaces and embedded within an extracellular polymeric substance (EPS) matrix. These biofilms form on various substrates, leading to associated infections in humans, plants, and animals (Lebeaux et al., 2014; Padmavathi et al., 2017; Kannappan et al., 2020). As demonstrated in Figure 3, CMRE significantly reduced biofilm formation of all test bacteria. Treatment with *CMRE* at ½ × MIC resulted in reduced biofilm formation in *P. aeruginosa* PAO1, *S. marcescens* MTCC 97, *C. violaceum* 12472, and *P. mirabilis* 425 by 69.1, 68.4, 76.9 and 70%, respectively, compared to their respective controls. Inhibition of biofilm in test bacterial strains was comparable with azithromycin (data not shown). The findings showed the potential of CMRE in controlling biofilm formation across various bacterial strains. Similar levels of biofilm inhibition have been reported for several plant extracts that inhibit biofilm formation in one or more pathogens (Famuyide et al., 2019; Alam et al., 2020; Siddiqui and Ahmad, 2024). Plant extracts contain a consortium of bioactive phytochemicals that often act synergistically, allowing them to target multiple stages of biofilm development. They can reduce adhesion-related gene expression, alter cell-surface hydrophobicity, chelate essential metal ions, and reduce EPS and disrupt cellular communication or QS, ultimately weakening biofilm structure and preventing its maturation (Vaou et al., 2021).

### 3.5 Reduction of exopolysaccharides production by *CMRE*

**FIGURE 4:**
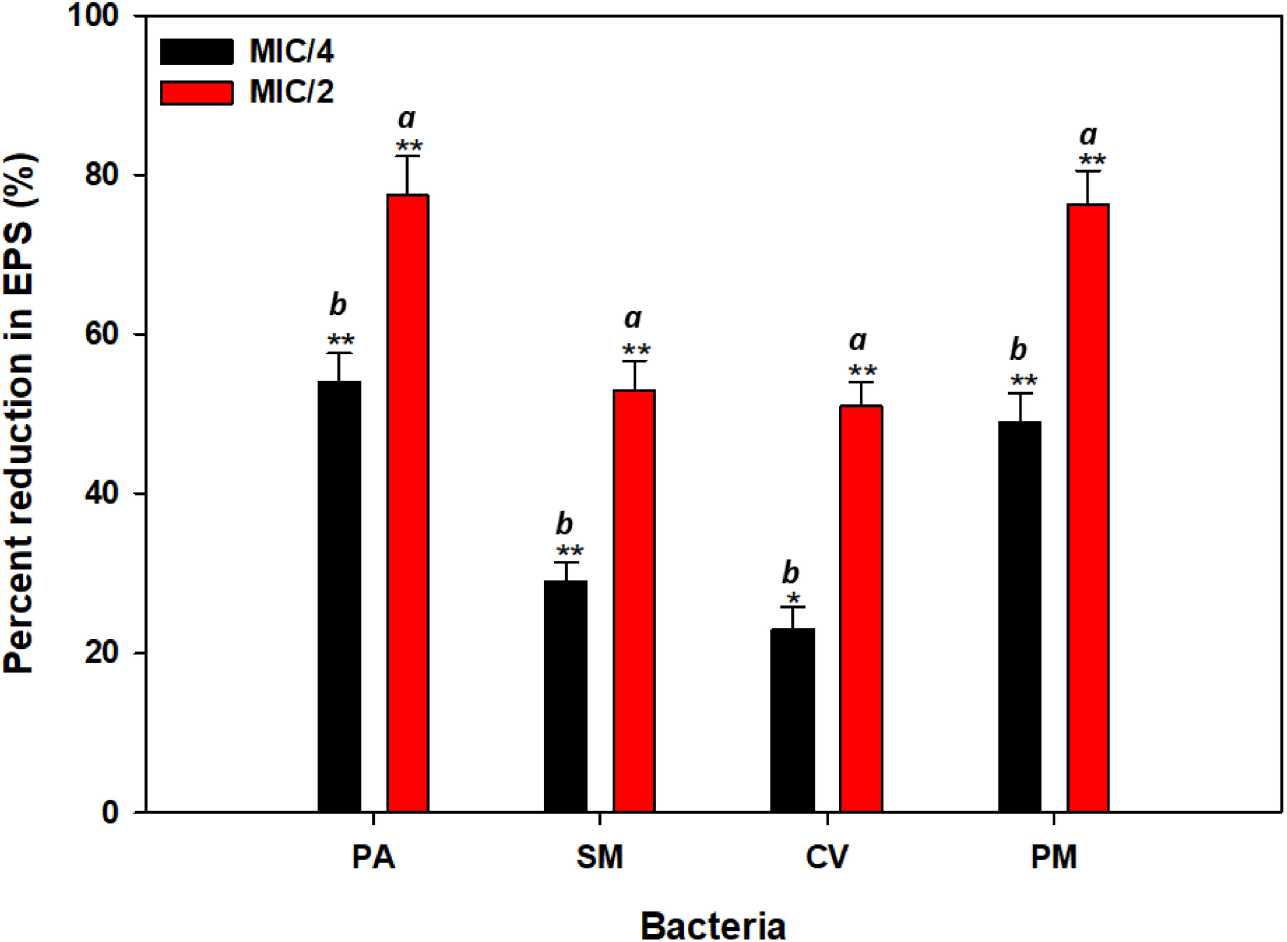
EPS inhibitory effect of sub-MICs of CMRE against bacterial pathogens. Data is presented as mean of triplicates and bar denotes SD. Different letters above the bars indicate statistically significant differences among treatments (*p* < 0.05). * depicts *p* ≤ 0.05 wrt to control and ** depicts *p* ≤ 0.01 wrt to control. Means denoted by the different letters within parameter are significantly different at *p*≤0.05.

Production of exopolysaccharides is crucial for biofilms, as it provides structural integrity and shields the biofilm from disruption by antimicrobial agents (Flemming and Wingender, 2010). By the treatment of CMRE, we observed reduced EPS production in all the tested pathogens (Figure 4). At the highest tested sub-MIC (½ × MIC) of CMRE, EPS production decreased by 77.4, 53, 51.6, and 76.3% in *P. aeruginosa* PAO1, *S. marcescens* MTCC 97, *C. violaceum* 12472, and *P. mirabilis* 425, respectively, compared to their respective untreated controls. The reduction in EPS production is likely to compromise biofilm architecture, making biofilms more susceptible to antimicrobials and facilitating their clearance by the host’s immune system (Rather et al., 2021b). Moreover, this approach highlights the potential of CMRE to inhibit biofilm formation by targeting key components like exopolysaccharide production, offering a promising avenue for combating biofilm-associated bacterial infections. Furthermore, as the current work is limited to bacterial systems, future studies will incorporate cytotoxicity assays in mammalian cells to verify the biocompatibility and therapeutic safety of CMRE.

### 3.6 Identification of major compounds in CMRE by GC/MS analysis

**FIGURE 5:**
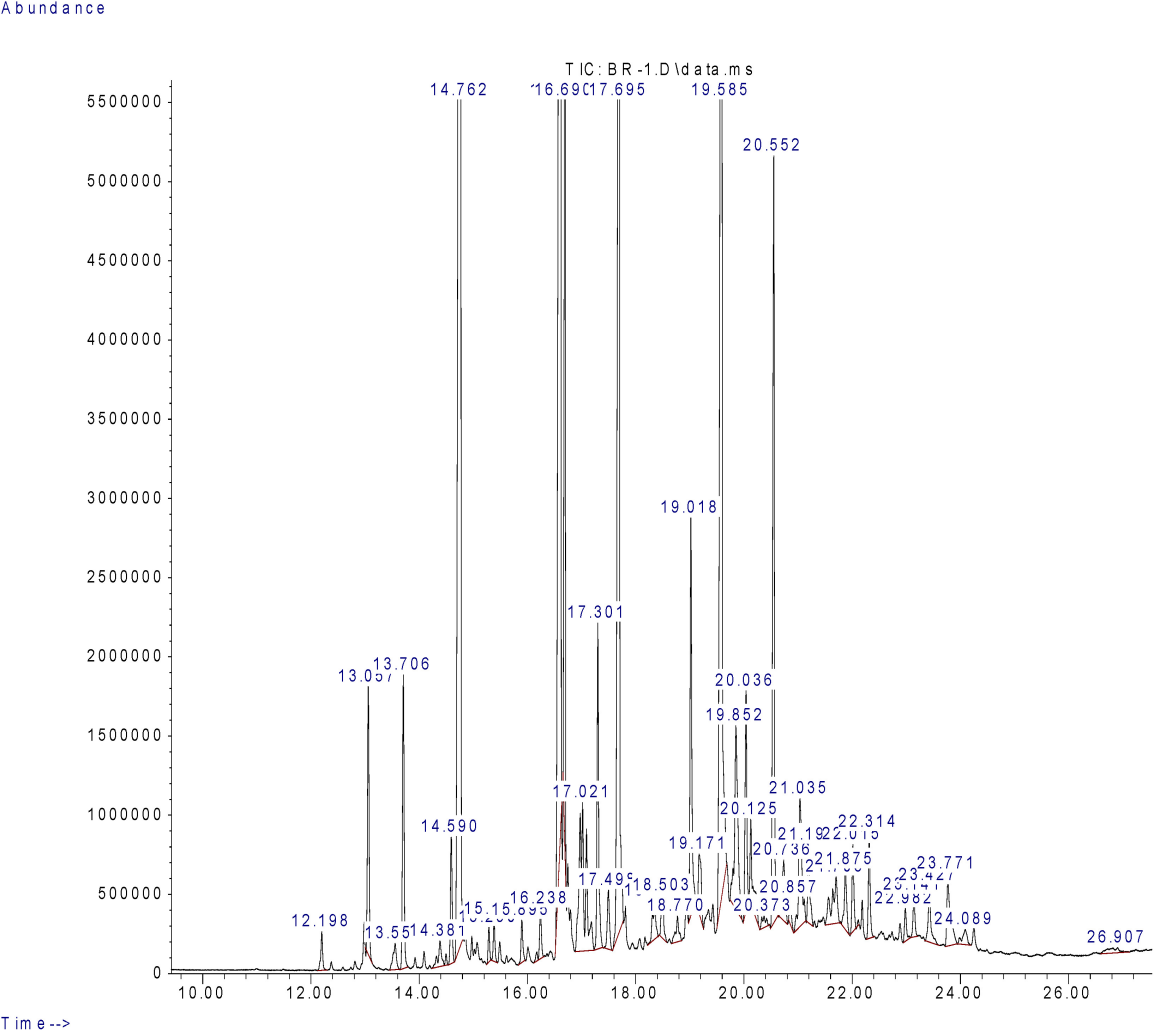
GC-MS chromatogram of CMRE.

For the identification of major phytocompounds present in CMRE, GC/MS analysis was performed. In Table 1, we present a list of phytochemicals detected in the methanolic extract of *C. myrrha* obtained using GC/MS. Additionally, Figure 5 displays the corresponding chromatogram. Among the identified compounds, curzerene was the most abundant, constituting 18.56% of the extract. Moreover, 4-(benzyloxy)aniline hydrochloride and 4-Methylimidazole-5-[1,1-dimethylethanol], 4-hydroxyphenyl(ether) was also detected in relatively larger amount. However, other compounds such as 2,6-diisopropylnaphthalene, β-elemene, and 2-ethyl-p-xylene were also detected using GC/MS in methanolic CMRE. Previously, the GC/MS analysis of essential oil of *C. myrrha* resin have found that α-elemene, 7-isopropyl-1,4-dimethyl-2-azulenol, curzerene, germacra-1(10)7,11-trien-15-oic acid,8,12-epoxy-6-hydroxy-ç-lactone, δ-elemene, δ-neoclovene, germacrene B, and eremophilene were the major phytocompounds with their abundances as 12.86, 12.22, 11.64, 6.20, 5.57, 5.57, 3.97, and 3.35%, respectively (Mohamed et al., 2014). Another study has also confirmed that the main composition of the essential oil of Ethiopian *C. myrrha* was furanoeudesma-1,3-diene (38.6%), curzerene (17.5%), lindestrene (14.4%), and α-elemene (4.3%) (Marongiu et al., 2005). Moreover, a report on phytochemical analysis of Iranian *C. myrrha* essential oil has found curzerene as the major phytocompound having more than 40% with its abundance (Morteza-Semnani and Saeedi, 2003). Quantitative predominance increases the likelihood of curzerene contributing to the observed biological effects of the CMRE. Although curzerene was identified as a major constituent of the methanolic CMRE by GC–MS, bioactivity-guided fractionation will be required to isolate it in pure form and confirm its specific contribution to the observed anti-QS and antibiofilm effects.

**TABLE 1:** Compounds detected by GC-MS analysis of CMRE.

RT (min)	Area (Ab*s)	Absolute height	Peak width	Compound name	Mol weight	Peak area (%)
14.76	37,703,407	11,127,860	0.159	Curzerene	216.1	18.56
16.60	31,193,080	10,500,341	0.146	4-Benzyloxyaniline hydrochloride	199.1	15.36
19.58	27,198,195	97,57,559	0.178	4-Benzyloxyaniline hydrochloride	199.1	13.39
17.69	2,48,49,524	9,420,767	0.191	4-Methylimidazole-5-[1,1-dimethylethanol], 4-hydroxyphenyl(ether)	246.1	12.24
20.55	10,059,197	5,161,542	0.146	2,6-Diisopropylnaphthalene	212.1	4.95
16.69	9,172,455	6,518,357	0.057	1,2-Ethanediol, 1,2-di-4-pyridinyl-	216.0	4.51
17.02	6,861,153	1,079,600	0.35	3,3’-Diamino-4,4’-biphenyldiol	216.0	3.37
19.01	5,478,028	2,874,961	0.134	4-Benzyloxyaniline	199.1	2.69
19.85	4,954,995	1,564,301	0.223	Methyl. alpha. -cyano-4-nitrocinnamate	232.0	2.43
17.30	4,578,346	2,213,238	0.159	4-Methylimidazole-5-[1,1-dimethylethanol], 4-hydroxyphenyl(ether)	246.1	2.25
13.70	4,051,541	1,885,048	0.146	Gamma. -Elemene	204.1	1.99
13.05	3,440,075	1,810,931	0.134	β-Elemene	204.1	1.69
21.03	3,354,960	1,104,408	0.223	2-Ethyl-p-xylene	134.1	1.65
20.03	3,136,392	1,784,192	0.108	(3S,4R,5R,6R)-4,5-Bis(hydroxymethyl)-3,6-dimethylcyclohexene	170.1	1.54
21.70	2,034,594	610,523	0.28	Ethanone, 1-[1-(4-amino-1,2,5-oxadiazol-3-yl)-4-methyl-1H-1,2,3-triazol-5-yl]-	208.0	1.00
23.77	1,974,414	629,811	0.223	2-Butanone, 4-(2,6,6-trimethyl-2-cyclohexen-1-ylidene)-	192.1	0.97
20.12	18,03,744	973,108	0.191	Acetamide, 2-hydrazino-2-oxo-N-(4-pentyloxyphenyl)-	265.1	0.88
14.59	1,791,479	860,550	0.121	Naphthalene, 1,2,3,4,4a,5,6,8a-octahydro-4a,8-dimethyl-2-(1-methylethenyl)-, [2R-(2.alpha.,4a.alpha.,8a.beta.)]-	204.1	0.88
20.73	1,591,587	715,763	0.172	Cyclopropane, 1-ethoxy-2,2-dimethyl-3-(2-phenylethenylidene)-	214.1	0.78
22.31	1,564,401	892,076	0.153	1-Cycloheptene, 1,4-dimethyl-3-(2-methyl-1-propene-1-yl)-4-vinyl-	204.1	0.77
22.01	1,349,681	814,949	0.159	Cyclohexane, 1,2-dimethyl-3,5-bis(1-methylethenyl)-, (1.alpha.,2.beta.,3.beta.,5.alpha.)-	192.1	0.66
19.17	1,314,563	753,586	0.146	Tricyclo[8.6.0.0(2,9)]hexadeca-3,15-diene, cis-2,9-anti-9,10-cis-1,10-	216.1	0.64
23.42	1,207,019	554,893	0.274	3-Buten-2-one, 4-(2,6,6-trimethyl-1-cyclohexen-1-yl)-, (E)-	192.1	0.59

Furthermore, its selection for *in silico* investigation serves as a rational mechanistic probe to help understand how key constituents of the extract may interact with QS and virulence regulatory proteins. Therefore, we have selected curzerene for further examination of in *in silico* studies.

### 3.7 Molecular docking of curzerene with the target protein involved in QS

To gain deeper insights into the interaction mechanisms between curzerene and the proteins associated with QS-mediated virulence factors, we conducted molecular modeling studies using AutoDock Vina. Curzerene was selected as a representative molecule due to its quantitative predominance in *C. myrrha* and because its potential role in QS and virulence regulation has not been characterized earlier. Therefore, the *in silico* analysis serves as a mechanistic model to illustrate how the major phytoconstituent of the extract may contribute to the observed anti-QS effects, rather than implying exclusivity of curzerene in the overall bioactivity.

Initially, we validated the docking procedure by extracting the antagonist (4-(3-bromophenoxy)-N-[(3S)-2-oxothiolan-3-yl] butanamide) from RhlR and then performing docking. Notably, our results showed that the ligand occupied the same position in the RhlR after docking as it did in the original crystal structure (Supplementary Figure 1). The binding energy for redocking of antagonist to RhlR was found to be –8.8 kcal/mol. The same docking procedure was applied for the docking of curzerene with the target proteins.

**FIGURE 6:**
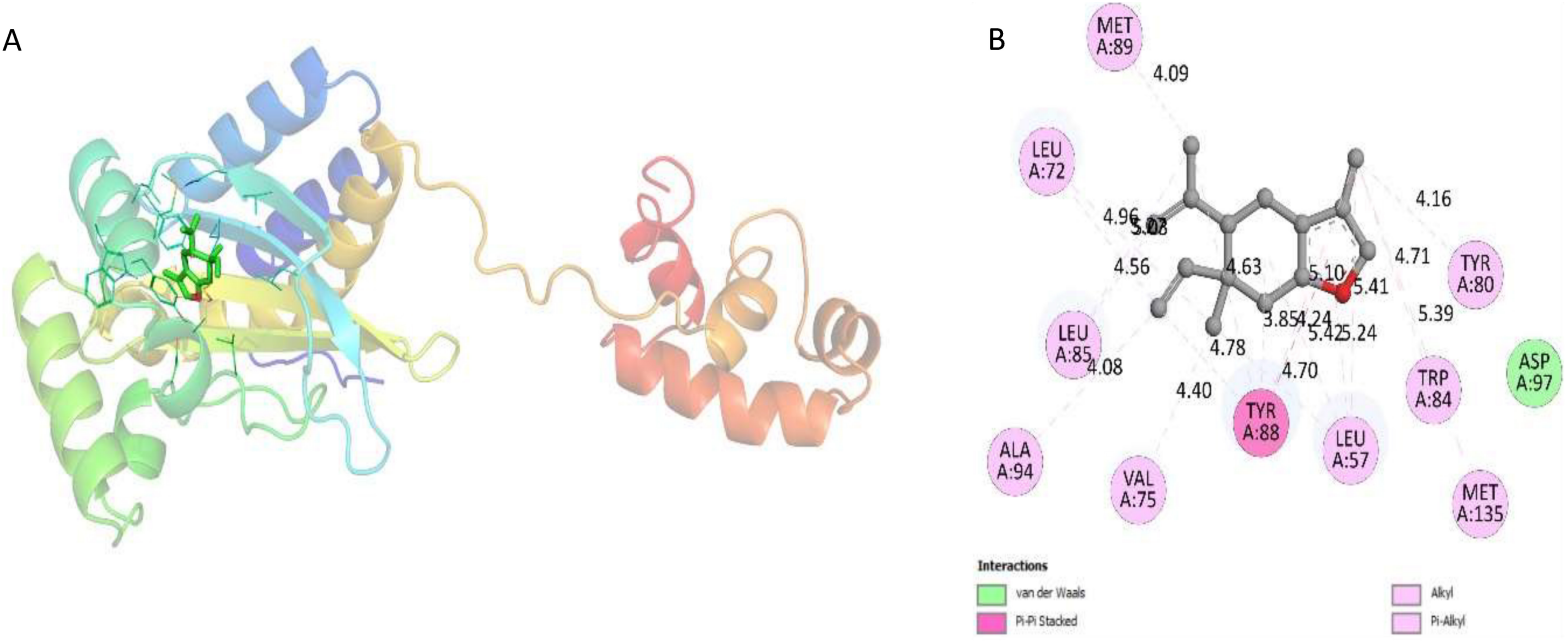
**(A)** Docked conformation of CviR-curzerene complex. CviR is shown as rainbow ribbons; curzerene is shown as green sticks. **(B)** Two-dimensional interaction diagram of CviR-curzerene complex.

In *C. violaceum* (ATCC 31532), the synthesis of violacein relies on QS, mediated by C_6_-AHL. This signaling molecule is detected by a LuxR-type protein known as CviR. CviR functions as a dimer, with two identical and overlapping subunits. Each monomer, comprising 250 amino acids, consists of two distinct domains: the DNA-binding domain (DBD) and the ligand-binding domain (LBD). The DBD is a smaller domain primarily composed of a few α-helices, while the LBD contains both α-helices and β-sheets. These two domains are connected by a short, flexible coil (Ahmed et al., 2013). Notably, the crystal structure of CviR reveals that the acyl group forms a hydrogen bond with Asp97, the lactone carbonyl forms another hydrogen bond with the conserved Trp84, and the carbonyl oxygen engages in two hydrogen bonds with Ser155 and Tyr80 (Ahmed et al., 2013). By molecular docking, we observed that curzerene was docked into the same cavity in CviR as its inhibitor, with a binding energy of –7.3 kcal/mol. Curzerene was surrounded by Met89, Leu72, Leu85, Ala94, Val75, Tyr88, Leu57, Met135, Trp84, and Tyr80 via hydrophobic forces (Figure 6). Moreover, Asp97 of CviR interacted with curzerene through van der Waals forces. Interestingly, C_8_-AHL and C_10_-AHL act as antagonists of the CviR receptor, while C_6_-AHL exhibits partial antagonist properties. A study has suggested an effective strategy to antagonize the transcription factor CviR using the compounds that bind to the receptor binding site of AHL autoinducers in *C. violaceum* (Chen et al., 2011). The docking studies suggest that curzerene may also possess antagonizing abilities, as it binds to the active site and potentially blocks the response of natural ligands to CviR.

**FIGURE 7:**
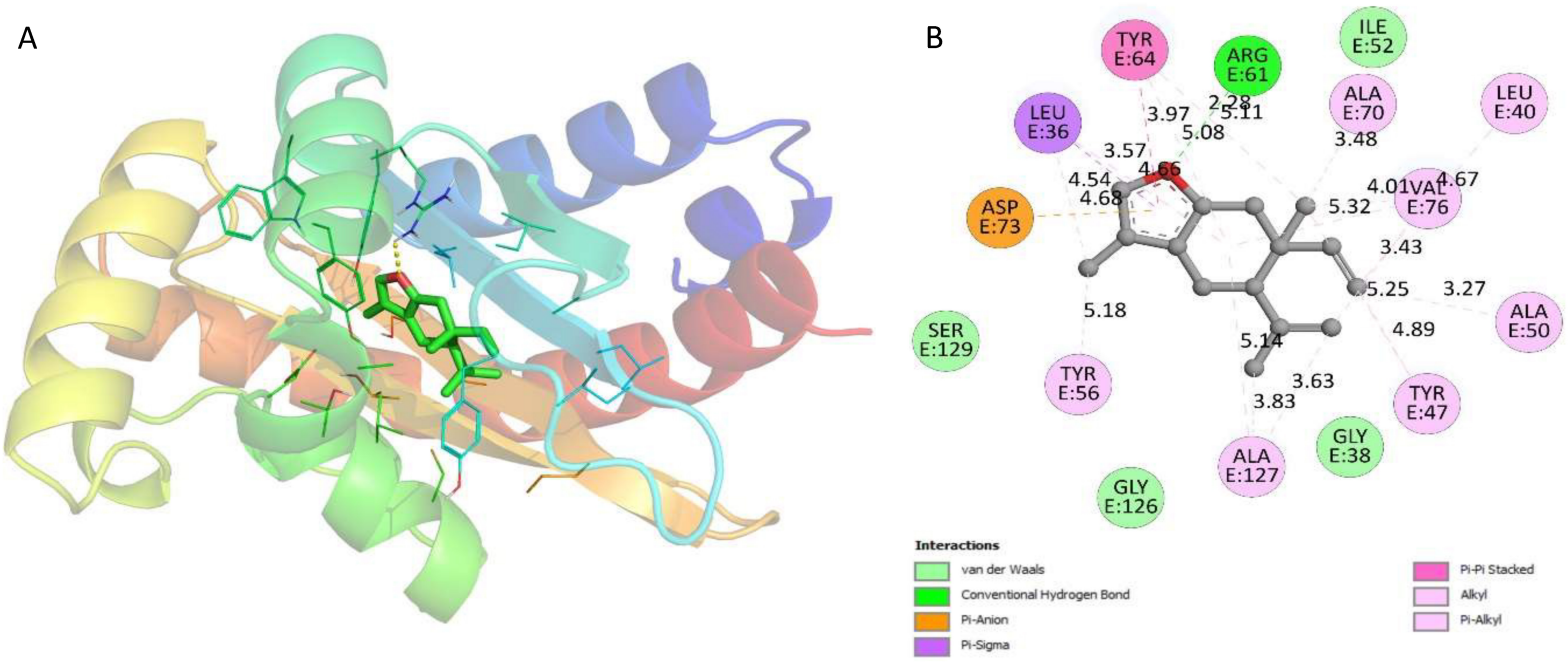
**(A)** Docked conformation of LasR-curzerene complex. LasR is shown as rainbow ribbons; curzerene is shown as green sticks. **(B)** Two-dimensional interaction diagram of LasR-curzerene complex.

The docking analysis of curzerene revealed that it exhibited remarkable affinity for LasR, with a predicted binding energy of –8.6 kcal/mol, corresponding to a binding constant of 2.03 × 10^6^ M^–1^. LasR. Curzerene interacted with Arg61 of LasR via one hydrogen bond, having a bond length of 2.28 Å (Figure 7). The complex was also stabilized by van der Waals forces (Ser129, Gly126, Gly38, and Ile52), hydrophobic interactions (Tyr56, Ala127, Tyr47, Ala50, Val76, Leu40, Ala70, Tyr64, and Leu36), and ionic forces (Asp73). When LasR combines with 3-oxo-C_12_HSL, it activates the expression of QS-regulated virulence genes in *P. aeruginosa* (Packiavathy et al., 2012). Interestingly, the competition between curzerene and 3-oxo-C_12_HSL for the same binding site could potentially lead to reduced production of QS-mediated factors (Yang et al., 2009). *In vitro* results from our study demonstrated that CMRE exhibited significant activity against LasR-dependent QS traits, including biofilm formation and motility in *P. aeruginosa* PAO1. The competitive binding of curzerene with key residues of target proteins may represent one of the mechanisms underlying their anti-QS effects.

**FIGURE 8:**
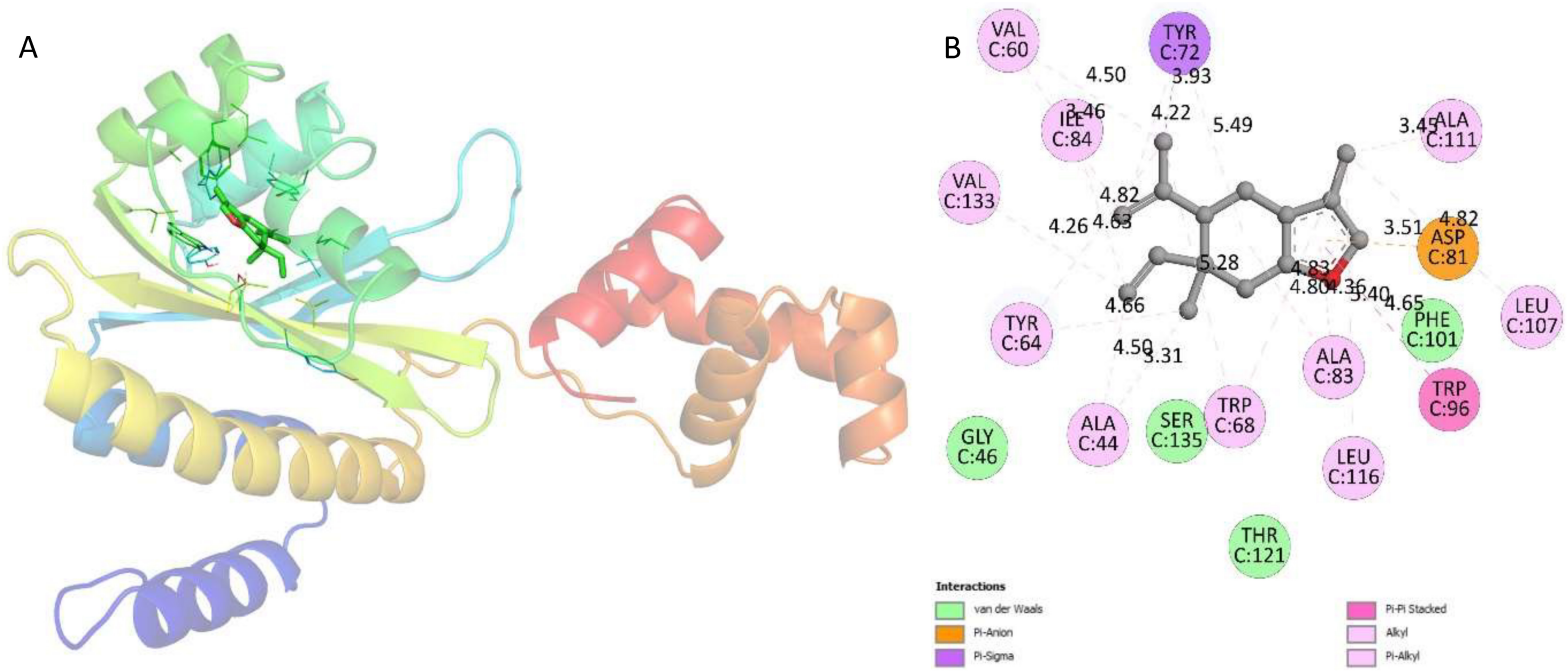
**(A)** Docked conformation of RhlR-curzerene complex. RhlR is shown as rainbow ribbons; curzerene is shown as green sticks. **(B)** Two-dimensional interaction diagram of RhlR-curzerene complex.

In *P. aeruginosa*, the transcription regulator RhlR plays a crucial role. When it complexes with butanoyl-homoserine lactone (C_4_-AHL), it activates the transcription of various virulence genes (Medina et al., 2003). Interestingly, curzerene demonstrated the binding energy of –6.5 kcal/mol toward RhlR. The residues that were involved in van der Waals interactions were Gly46, Ser135, Thr121, and Phe101 (Figure 8). Moreover, there were also involvement of hydrophobic forces (Trp68, Ala83, Leu116, Trp96, Leu107, Ala111, Tyr72, Ile84, Val60, Val133, Tyr164, and Ala44) and ionic forces (Asp81) in the complexation of curzerene with RhlR. Docking investigations also revealed that curzerene occupied the same binding cavity where its inhibitor (4-(3-bromophenoxy)-N-[(3S)-2-oxothiolan-3-yl] butanamide) binds. These findings are consistent with previous reports demonstrating that plant-derived phytocompounds can effectively interact with QS-related regulatory proteins and attenuate QS-mediated virulence (Qais et al., 2024; Al-Shabib et al., 2025). Since, curzerene interacted with key residues of target proteins that are also affected by furanone-based inhibitors (Zou and Nair, 2009; Ravichandran et al., 2018). This suggests that the binding orientation is similar and that there may be a mechanism for competitive inhibition. This overlap in binding residues may make it harder for the natural ligand to get to its target, which would weaken QS signaling and the expression of virulence downstream. Curzerene, with its natural origin and stable binding to QS regulators, could be a structural lead for non-antibiotic antivirulence agents targeting Gram-negative pathogens.

*In silico* findings support the idea that phytoconstituents of CMRE bind to QS receptor proteins, effectively blocking the attachment of signal molecules to these receptors. As a result, QS-dependent virulence factors are inhibited, preventing pathogens from accurately sensing their own cell.

### 3.8 Molecular dynamics simulations

**FIGURE 9:**
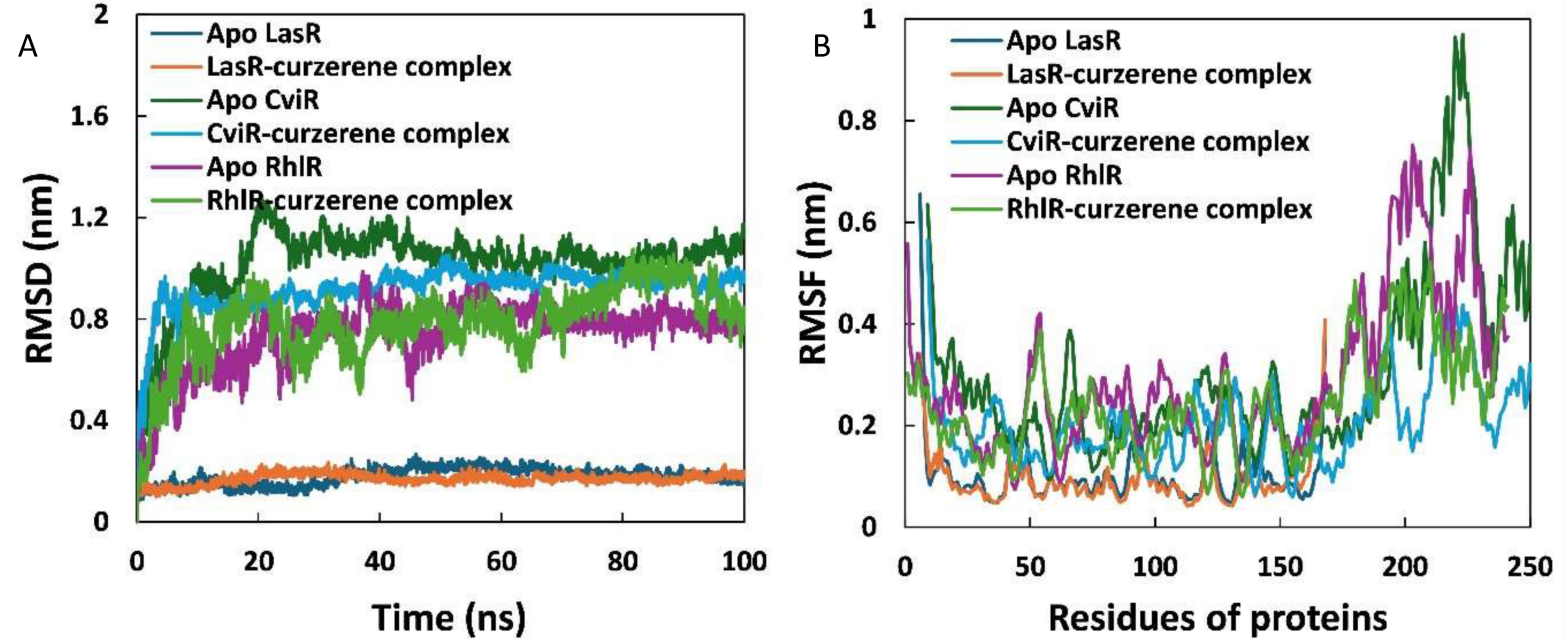
**(A)** Root mean square deviations (RMSD) of apo LasR, LasR-curzerene complex, apo CviR, CviR-curzerene complex, apo RhlR, and RhlR-curzerene complex over 100 ns MD simulation. **(B)** Root mean square fluctuations (RMSF) of LasR, CviR, and RhlR in absence and presence of curzerene.

Three proteins, each targeting different aspects of QS, were selected for further study by simulating them in a physiological environment. The docked complexes with the lowest binding energies were chosen as the starting conformations for MD simulations. To preliminarily assess the stability of these complexes, root mean square deviations (RMSDs) of backbone atoms were calculated relative to their initial structures (Cui et al., 2015). Figure 9A shows the RMSDs for LasR, RhlR, and CviR, both alone and in complex with curzerene. Throughout the simulation period, the RMSDs for LasR and its complex remained below 0.2 nm. The RMSD of the other two proteins (RhlR and CviR) and its complexes with curzerene reached a plateau roughly before 40 ns of simulation. Additionally, there was a minimal difference in the average RMSDs of the apo proteins compared to their respective complexes. For example, the average RMSD for LasR and LasR-curzerene complex were 0.177 and 0.169 nm, respectively. These findings indicate the stable nature of the complexes in an aqueous environment. Similar results have been reported previously, where the average RMSD values for the LasR-bakuchiol complex ranged from 0.20 to 0.30 nm (Husain et al., 2018).

The root mean square fluctuation (RMSF) of the C_α_ atoms for each residue of LasR, RhlR, and CviR in the absence and presence of curzerene was also calculated, with the results shown in Figure 9B. The RMSF values for nearly all amino acids were below 0.3 nm. Additionally, the fluctuation patterns of the residues in all three proteins in the presence of curzerene were similar to those of the apo proteins, with only minor deviations. For instance, the average RMSF of all residues of LasR in the absence and presence of curzerene was 0.104 and 0.093 nm, respectively. Similarly, the complexation of curzerene to the other two proteins (RhlR and CviR) also decreased the average fluctuation of the residues. This RMSF analysis further supports the stable nature of the complexes under physiological conditions.

**FIGURE 10:**
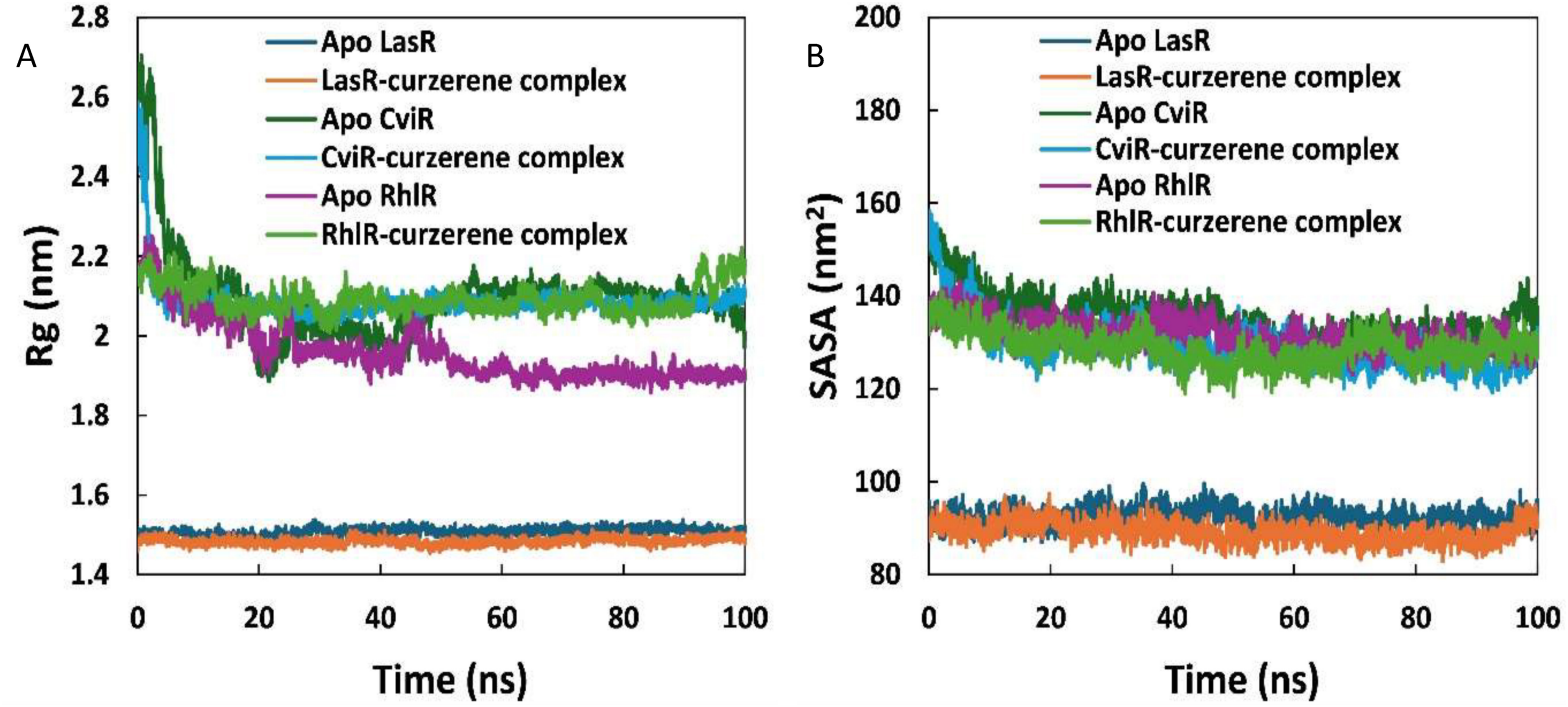
**(A)** Radius of gyration (Rg) of apo LasR, LasR-curzerene complex, apo CviR, CviR-curzerene complex, apo RhlR, and RhlR-curzerene complex over 100 ns MD simulation. **(B)** Solvent accessible surface area (SASA) of apo LasR, LasR-curzerene complex, apo CviR, CviR-curzerene complex, apo RhlR, and RhlR-curzerene complex over 100 ns MD simulation.

The radius of gyration (R_g_) is defined as the mass-weighted root mean square distance of a collection of atoms from their common center of mass (Lobanov et al., n.d.). It serves as an indicator of the stability of complexes during MD simulations. Figure 10A shows the R_g_ values for the simulated proteins and their complexes with curzerene. The R_g_ trajectory of the complexes was identical to that of the respective apo proteins, with minimal differences observed over the simulation period. For instance, the average R_g_ of apo LasR and LasR-curzerene complex was 1.507 and 1.483 nm, respectively. Similarly, the average R_g_ of apo CviR and CviR-curzerene complex was found as 2.093 and 2.086 nm, respectively. Additionally, the solvent accessible surface area (SASA) of the apo proteins and protein-curzerene complexes was calculated, as shown in Figure 10B. The SASA values of the complexes were insignificantly different from those of the respective apo proteins. For example, the SASA of apo LasR and LasR-curzerene complex were 92.372 and 89.214 nm^2^, respectively. The SASA of complexes of curzerene with the other two proteins (RhlR and CviR) were also roughly the same compared to their apo proteins. The SASA remained constant throughout the simulation for all simulations. The analysis of R_g_ and SASA indicates that there were negligible structural changes, further confirming their stability.

**FIGURE 11:**
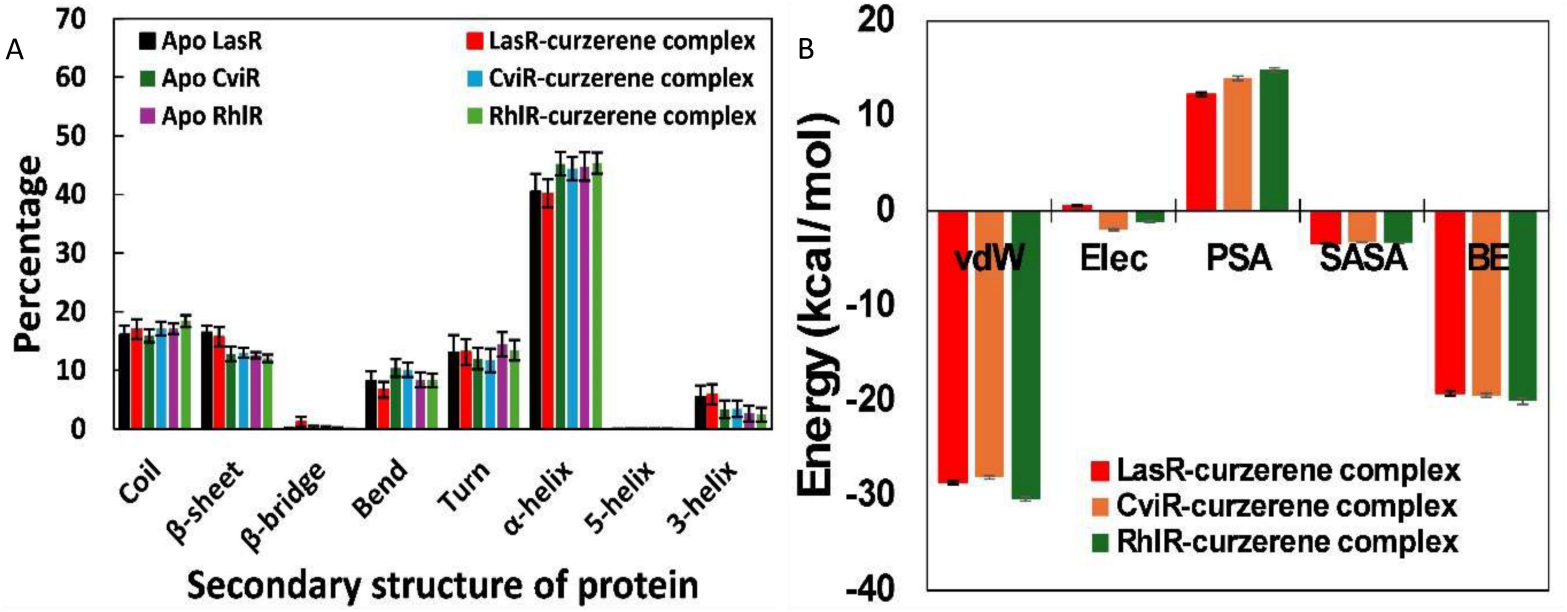
**(A)** Average percentage of secondary structures in LasR, CviR, and RhlR in absence and presence of curzerene. **(B)** MM-PBSA binding energies for the interaction of curzerene with LasR, CviR, or RhlR.

The impact of curzerene on the secondary structures of the tested proteins was evaluated by analyzing their secondary structure components, as shown in Figure 11A. The differences in each secondary structural element in the absence and presence of curzerene were minimal. For instance, the α-helix content in apo LasR was 40.506%, which remained nearly unchanged at 40.194% in the LasR-curzerene complex. This finding aligns with previous reports, where the α-helix content in LasR was found to be 41.15% (Qais et al., 2023). Similarly, the α-helical content in RhlR and its complex with curzerene was 44.758 and 45.354%, respectively. Comparable results were observed for other secondary motifs in CviR, indicating the structural stability of the complexes in a physiological environment.

To understand the forces involved in the interaction between curzerene and proteins, MM-PBSA analysis was conducted. This analysis involved extracting xx snapshots from the entire simulation trajectory of all three complexes. Typically, protein-ligand complexes are stabilized by non-covalent forces (Siddiqui et al., 2019). Some of these forces enhance the overall binding, while others may impede it. Van der Waals forces were the primary contributors to the binding interactions with all three proteins (Figure 11B). Additionally, there were minor contributions from solvent-accessible surface area (SASA) energies. The polar solvation energy was found to be positive which in unfavorable. The polar solvation energy hindered the overall binding of curzerene to the target proteins. The average binding energies for curzerene with LasR, CviR, and RhlR were calculated to be –19.230, –19.444, and –20.052 kcal/mol, respectively.

The MM-PBSA data was further used for the calculation energy contribution of residues of the proteins. The contribution of individual residues to the overall binding was also calculated, with the residues showing the highest energy contributions are discussed below. In the binding of curzerene to LasR, the highest energy contribution came from Ile52 (–1.466 ± 0.043 kcal/mol) followed by Tyr64 (–0.952 ± 0.054 kcal/mol), Leu36 (–0.917 ± 0.037 kcal/mol), Val76 (–0.561 ± 0.021 kcal/mol), Phe37 (–0.455 ± 0.018 kcal/mol), Ala50 (–0.399 ± 0.024 kcal/mol), Ala127 (–0.382 ± 0.037 kcal/mol), Pro57 (–0.311 ± 0.010 kcal/mol), Gly38 (–0.303 ± 0.027 kcal/mol), Phe51 (–0.285 ± 0.012 kcal/mol), Leu39 (–0.271 ± 0.011 kcal/mol), and Ala70 (–0.257 ± 0.013 kcal/mol), as shown in Table 2. In the CviR and curzerene binding, Tyr88 (–1.892 ± 0.061 kcal/mol) emerged as highest energy contributor followed by Leu57 (–1.025 ± 0.037 kcal/mol), Met135 (–0.890 ± 0.031 kcal/mol), Leu85 (–0.825 ± 0.028 kcal/mol), Ile99 (–0.649 ± 0.032 kcal/mol), Leu100 (–0.558 ± 0.027 kcal/mol), Ile153 (–0.482 ± 0.024 kcal/mol), Trp111 (–0.421 ± 0.039 kcal/mol), Tyr80 (–0.411 ± 0.040 kcal/mol), Leu72 (–0.354 ± 0.023 kcal/mol), Phe126 (–0.313 ± 0.021 kcal/mol), and Val75 (–0.243 ± 0.021 kcal/mol) (Table 2). The energy decomposition of RhlR-curzerene complex found that Tyr72 (–1.521 ± 0.060 kcal/mol) exhibited highest energy in overall binding energy, as presented in Table 2, followed by Val60 (–0.610 ± 0.054 kcal/mol), Ala44 (–0.606 ± 0.040 kcal/mol), Leu69 (–0.496 ± 0.030 kcal/mol), Val133 (–0.437 ± 0.041 kcal/mol), Tyr64 (–0.431 ± 0.024 kcal/mol), Trp96 (–0.355 ± 0.042 kcal/mol), Ile84 (–0.329 ± 0.028 kcal/mol), Leu107 (–0.269 ± 0.028 kcal/mol), Gly46 (–0.243 ± 0.024 kcal/mol), Glu59 (–0.243 ± 0.014 kcal/mol), and Ala83 (–0.239 ± 0.012 kcal/mol).

**TABLE 2:** Total energies of major energy contributing residues of LasR, CviR, and RhlR for the interaction with curzerene.

QS proteins					
LasR		CviR		RhlR	
Residues	Total energy	Residues	Total energy	Residues	Total energy
Leu36	–0.917 ± 0.037	Leu57	–1.025 ± 0.037	Ala44	–0.606 ± 0.040
Phe37	–0.455 ± 0.018	Leu72	–0.354 ± 0.023	Gly46	–0.243 ± 0.024
Gly38	–0.303 ± 0.027	Val75	–0.243 ± 0.021	Glu59	–0.243 ± 0.014
Leu39	–0.271 ± 0.011	Tyr80	–0.411 ± 0.040	Val60	–0.610 ± 0.054
Ala50	–0.399 ± 0.024	Leu85	–0.825 ± 0.028	Tyr64	–0.431 ± 0.024
Phe51	–0.285 ± 0.012	Tyr88	–1.892 ± 0.061	Leu69	–0.496 ± 0.030
Ile52	–1.466 ± 0.043	Ile99	–0.649 ± 0.032	Tyr72	–1.521 ± 0.060
Pro57	–0.311 ± 0.010	Leu100	–0.558 ± 0.027	Ala83	–0.239 ± 0.012
Tyr64	–0.952 ± 0.054	Trp111	–0.421 ± 0.039	Ile84	–0.329 ± 0.028
Ala70	–0.257 ± 0.013	Phe126	–0.313 ± 0.021	Trp96	–0.355 ± 0.042
Val76	–0.561 ± 0.021	Met135	–0.890 ± 0.031	Leu107	–0.269 ± 0.028
Ala127	–0.382 ± 0.037	Ile153	–0.482 ± 0.024	Val133	–0.437 ± 0.041

**FIGURE 12:**
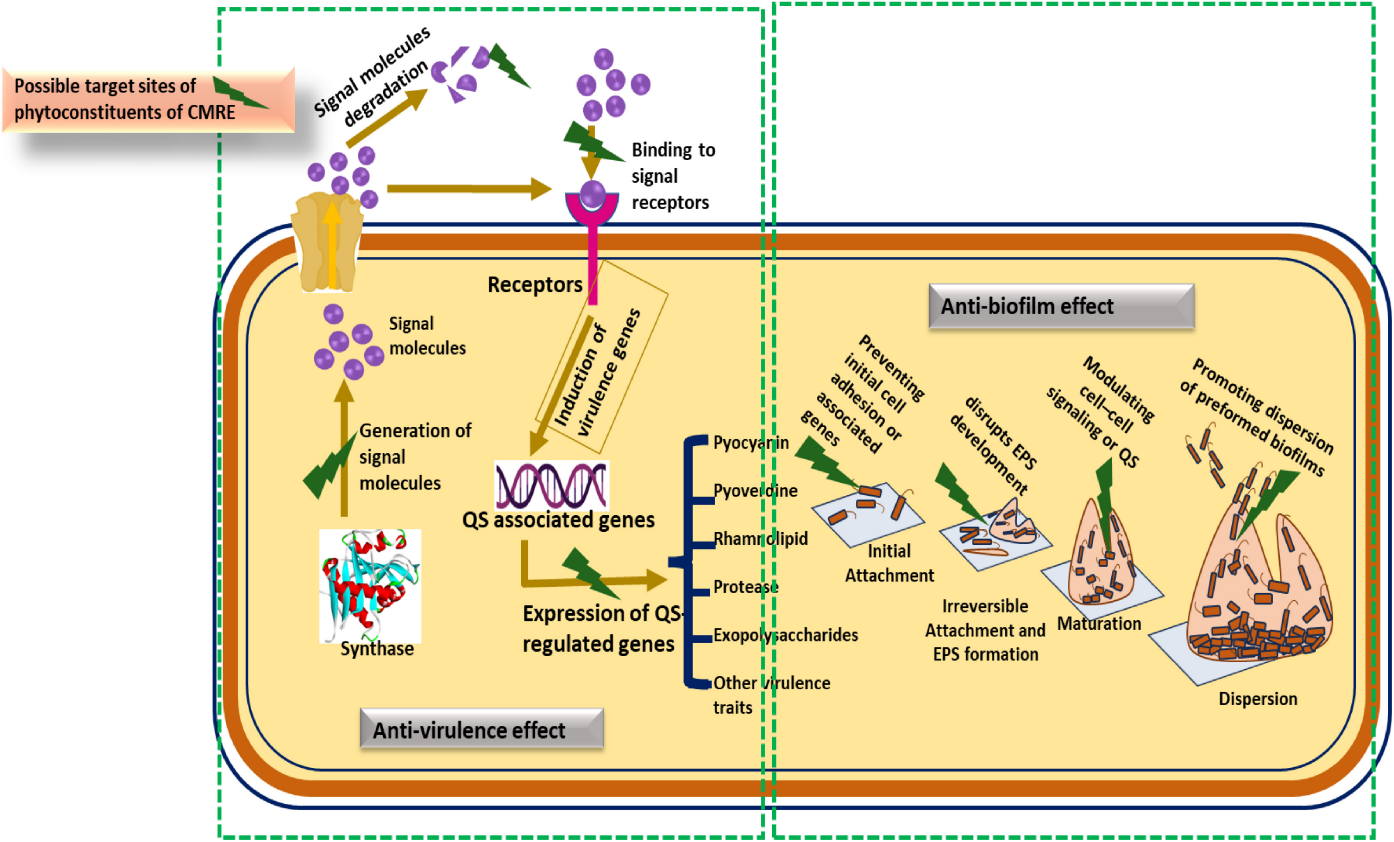
Possible mode of action phytoconstituents of CMRE for virulence and biofilm inhibition.

The overall proposed mechanism through which CMRE and curzerene interfere with QS signaling and biofilm formation is summarized in Figure 12.

## 4 Conclusion

This study explored the impact of *Commiphora myrrha* on the virulence and biofilm formation of Gram-negative bacteria. The extract significantly reduced violacein production in *C. violaceum* 12472 and decreased pyocyanin, pyoverdin, and rhamnolipid production in P. *aeruginosa* PAO1. CSH of test pathogens also decreased in the presence of CMRE. Treatment with the extract led to reduced biofilm formation in various Gram-negative strains. Further evaluation in mixed-species biofilm systems will clarify whether CMRE’s effects extend to more complex microbial communities. Curzerene, the most abundant phytoconstituent, showed promising interactions with key proteins involved in quorum sensing. These findings suggest that *C. myrrha* could be a valuable source for new anti-virulence drugs against Gram-negative bacteria.

## Data Availability

The raw data supporting the conclusions of this article will be made available by the authors, without undue reservation.
