# Supplementary material for: Commiphora myrrha resin extract inhibits the biofilms and quorum sensing controlled virulence factors of Gram-negative foodborne bacterial pathogens
Source: Front Microbiol. 2025 Dec 31;16:1668863. doi: 10.3389/fmicb.2025.1668863 (PMC12801156; doi:10.3389/fmicb.2025.1668863)
Supplement: Supplementary file 1 [file Presentation_1.PPTX]

## Slide 1
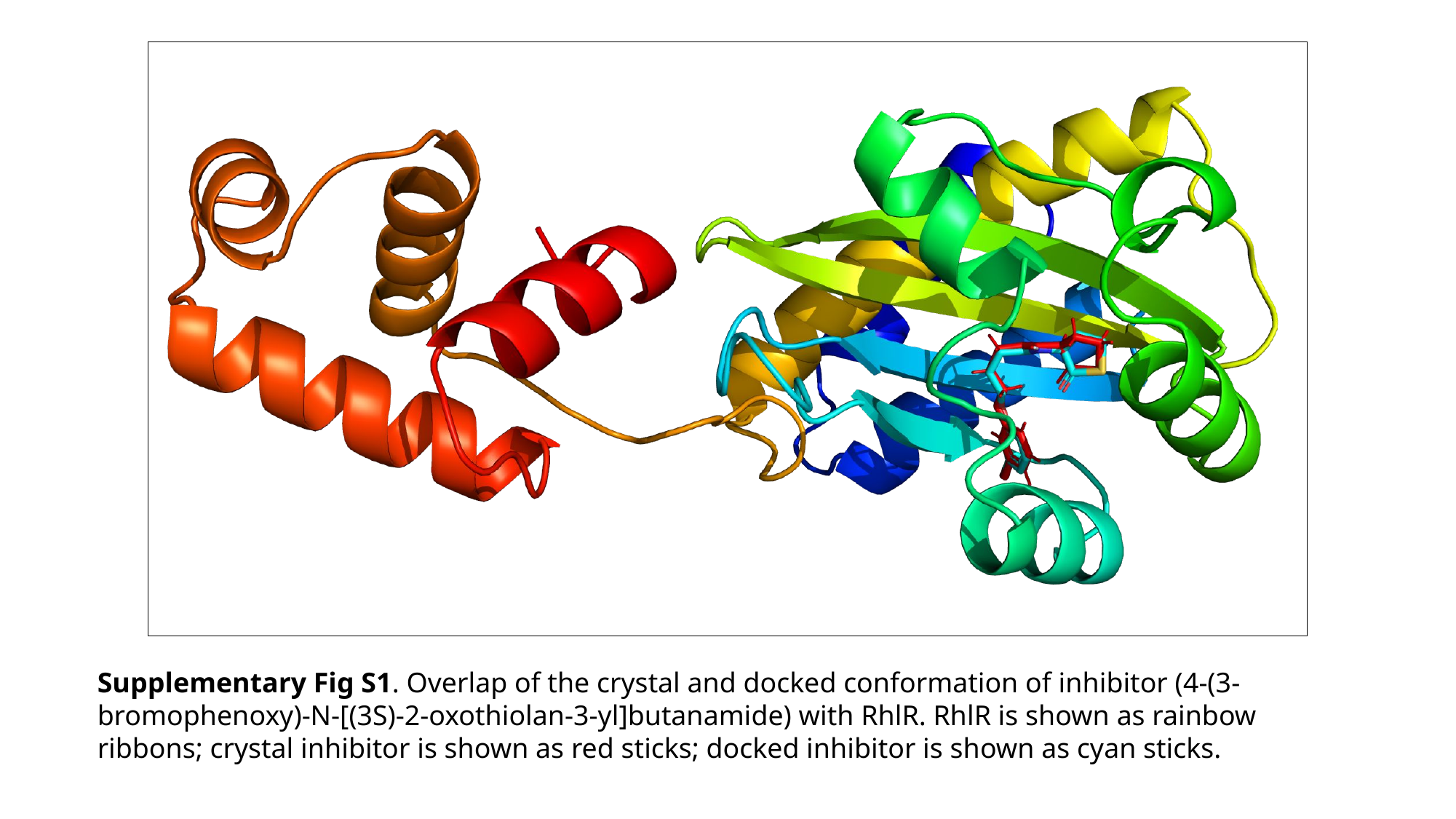

Supplementary Fig S1. Overlap of the crystal and docked conformation of inhibitor (4-(3-bromophenoxy)-N-[(3S)-2-oxothiolan-3-yl]butanamide) with RhlR. RhlR is shown as rainbow ribbons; crystal inhibitor is shown as red sticks; docked inhibitor is shown as cyan sticks.

## Slide 2
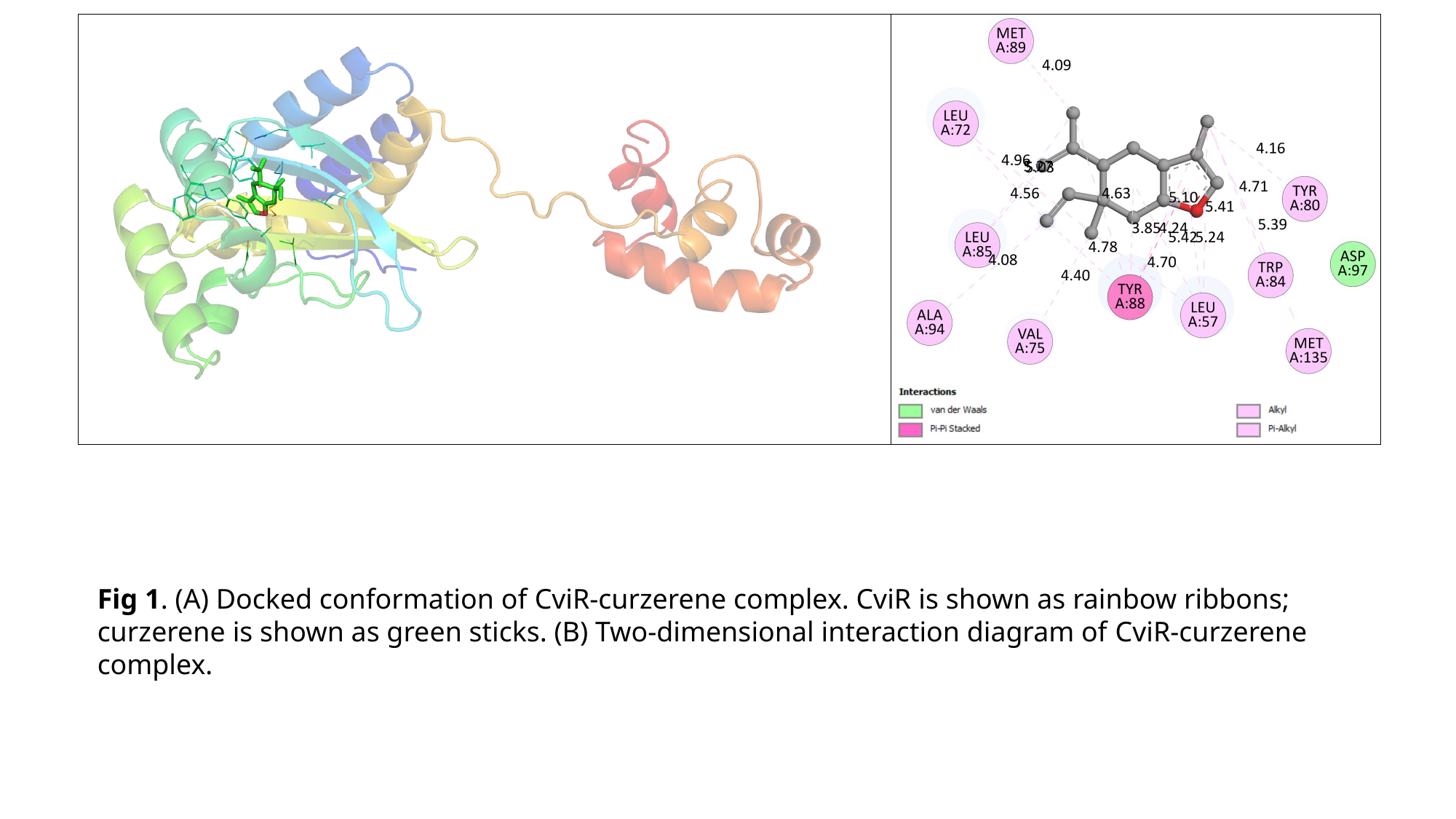

Fig 1. (A) Docked conformation of CviR-curzerene complex. CviR is shown as rainbow ribbons; curzerene is shown as green sticks. (B) Two-dimensional interaction diagram of CviR-curzerene complex.

## Slide 3
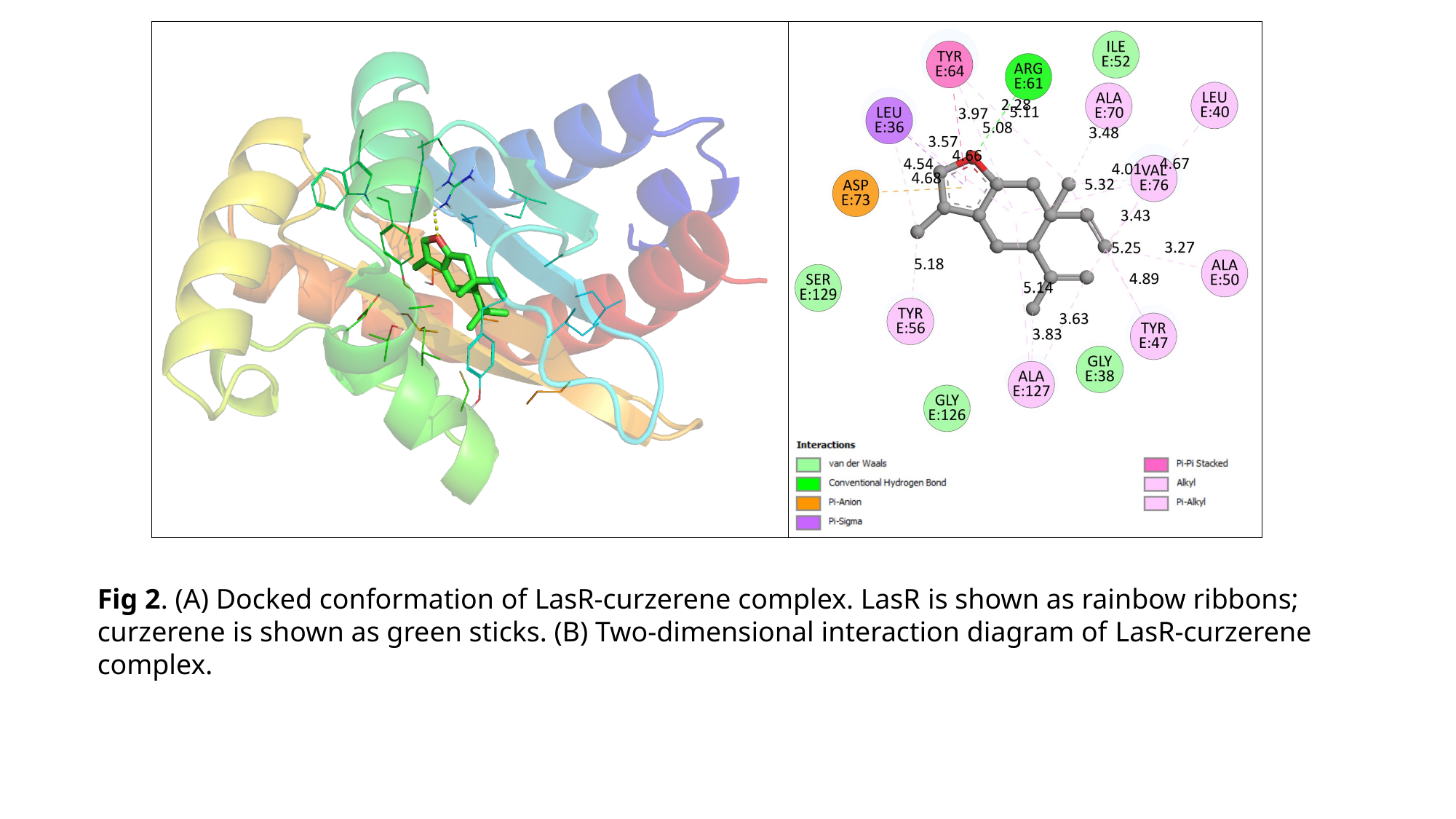

Fig 2. (A) Docked conformation of LasR-curzerene complex. LasR is shown as rainbow ribbons; curzerene is shown as green sticks. (B) Two-dimensional interaction diagram of LasR-curzerene complex.

## Slide 4
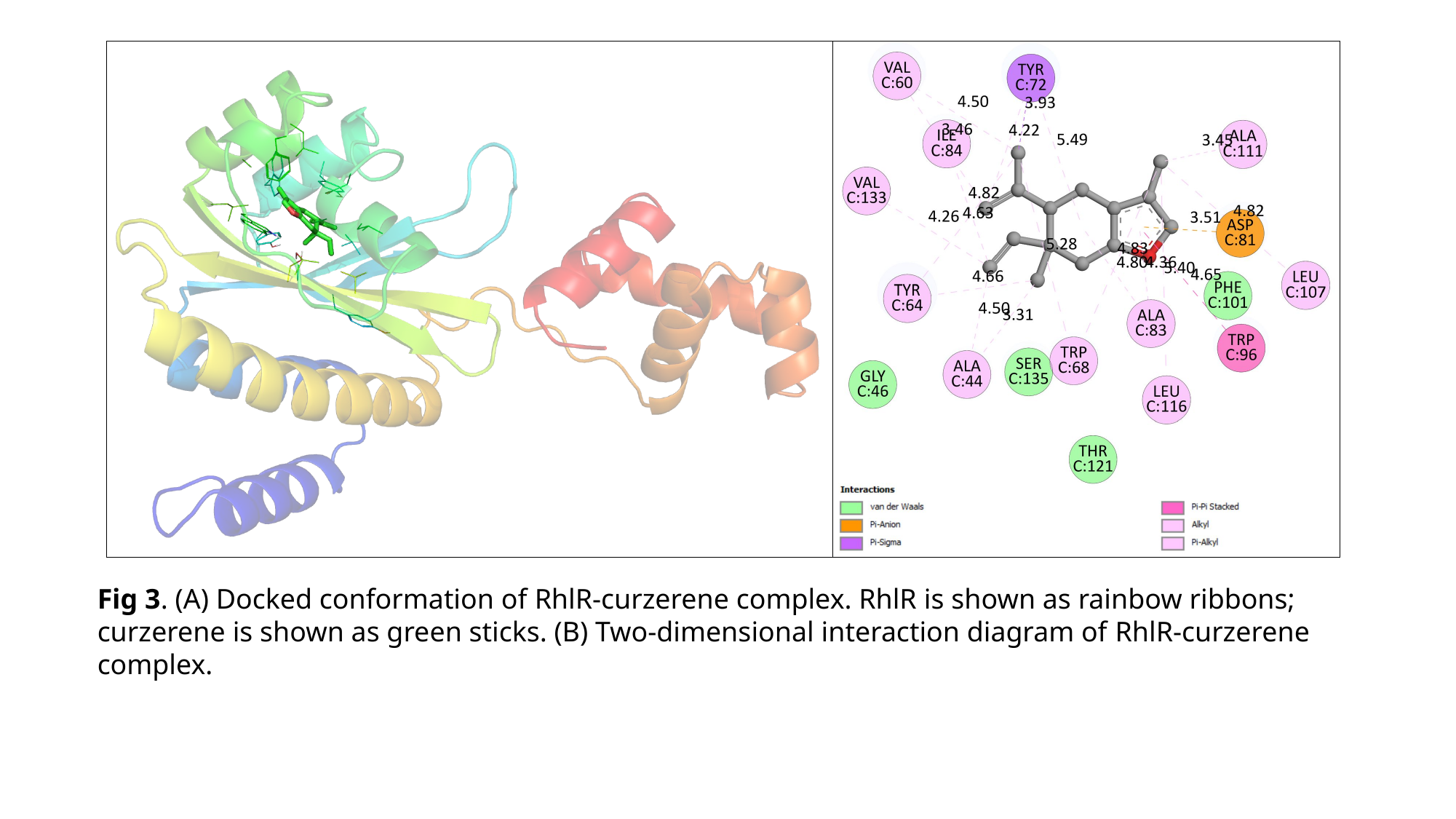

Fig 3. (A) Docked conformation of RhlR-curzerene complex. RhlR is shown as rainbow ribbons; curzerene is shown as green sticks. (B) Two-dimensional interaction diagram of RhlR-curzerene complex.

## Slide 5
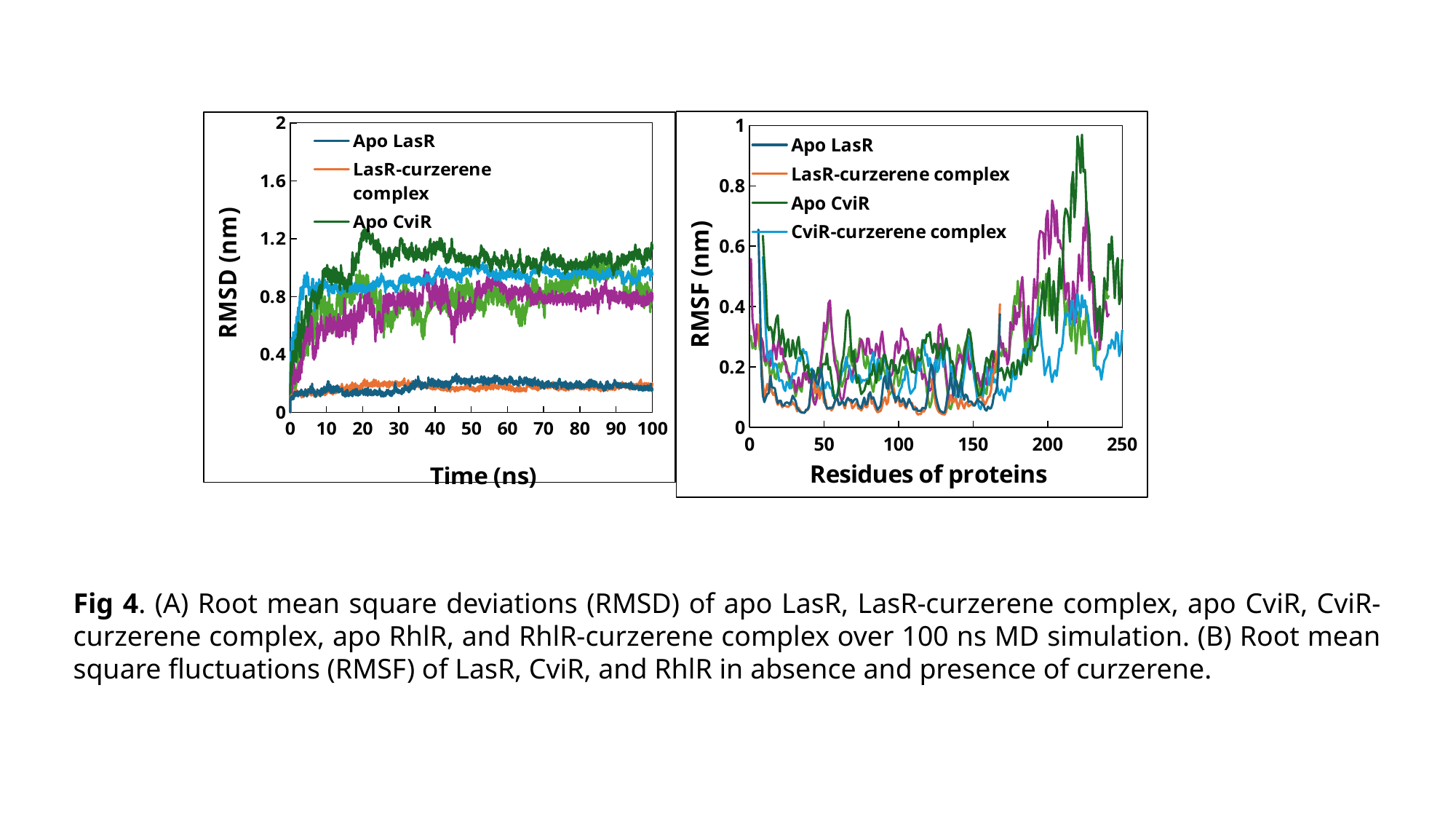

### Chart
| Category | Apo LasR | LasR-curzerene complex | Apo CviR | CviR-curzerene complex | Apo RhlR | RhlR-curzerene complex |
|---|---|---|---|---|---|---|
### Chart
| Category | Apo LasR | LasR-curzerene complex | Apo CviR | CviR-curzerene complex | Apo RhlR | RhlR-curzerene complex |
|---|---|---|---|---|---|---|Fig 4. (A) Root mean square deviations (RMSD) of apo LasR, LasR-curzerene complex, apo CviR, CviR-curzerene complex, apo RhlR, and RhlR-curzerene complex over 100 ns MD simulation. (B) Root mean square fluctuations (RMSF) of LasR, CviR, and RhlR in absence and presence of curzerene.

## Slide 6
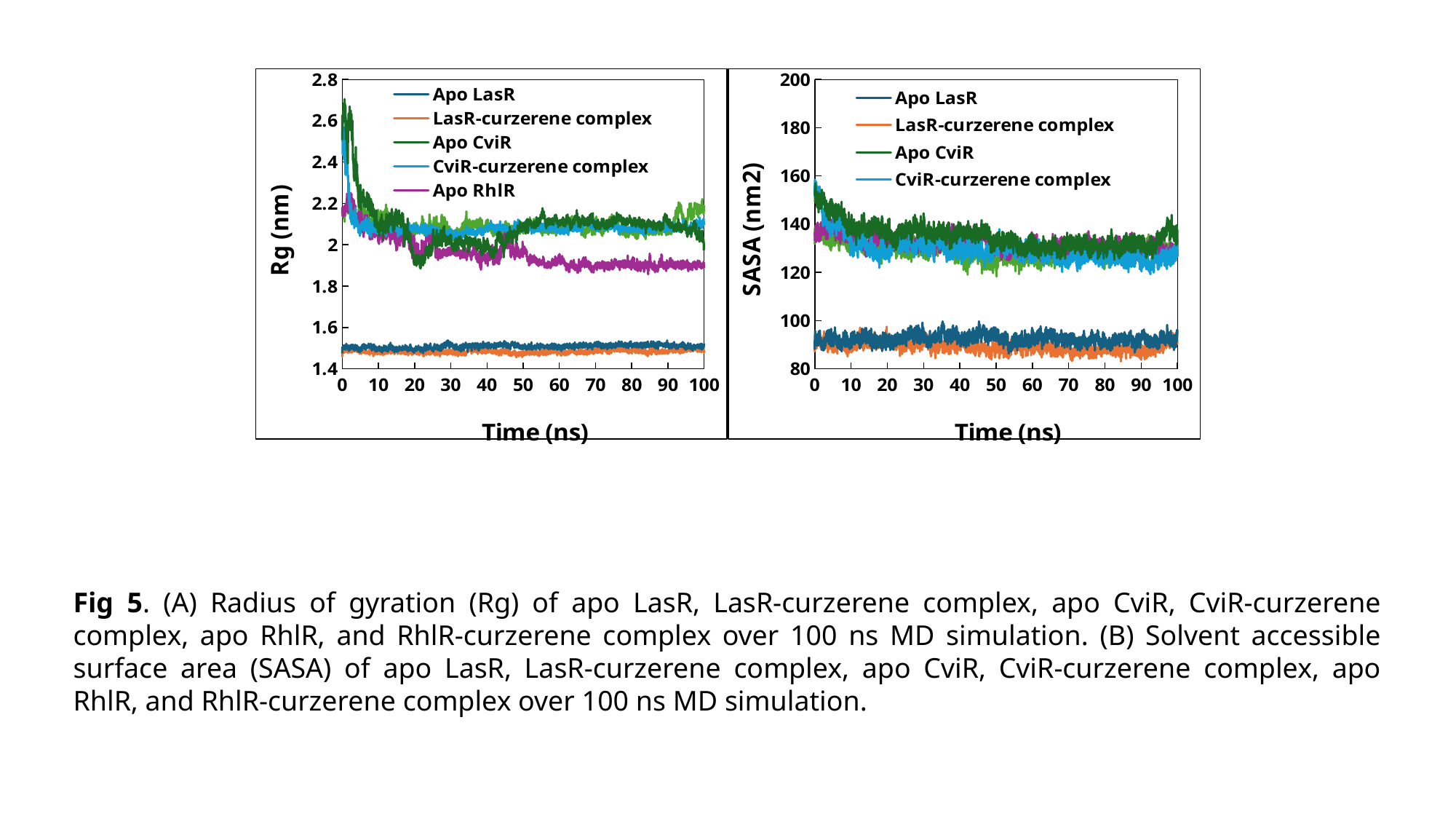

### Chart
| Category | Apo LasR | LasR-curzerene complex | Apo CviR | CviR-curzerene complex | Apo RhlR | RhlR-curzerene complex |
|---|---|---|---|---|---|---|
### Chart
| Category | Apo LasR | LasR-curzerene complex | Apo CviR | CviR-curzerene complex | Apo RhlR | RhlR-curzerene complex |
|---|---|---|---|---|---|---|Fig 5. (A) Radius of gyration (Rg) of apo LasR, LasR-curzerene complex, apo CviR, CviR-curzerene complex, apo RhlR, and RhlR-curzerene complex over 100 ns MD simulation. (B) Solvent accessible surface area (SASA) of apo LasR, LasR-curzerene complex, apo CviR, CviR-curzerene complex, apo RhlR, and RhlR-curzerene complex over 100 ns MD simulation.

## Slide 7
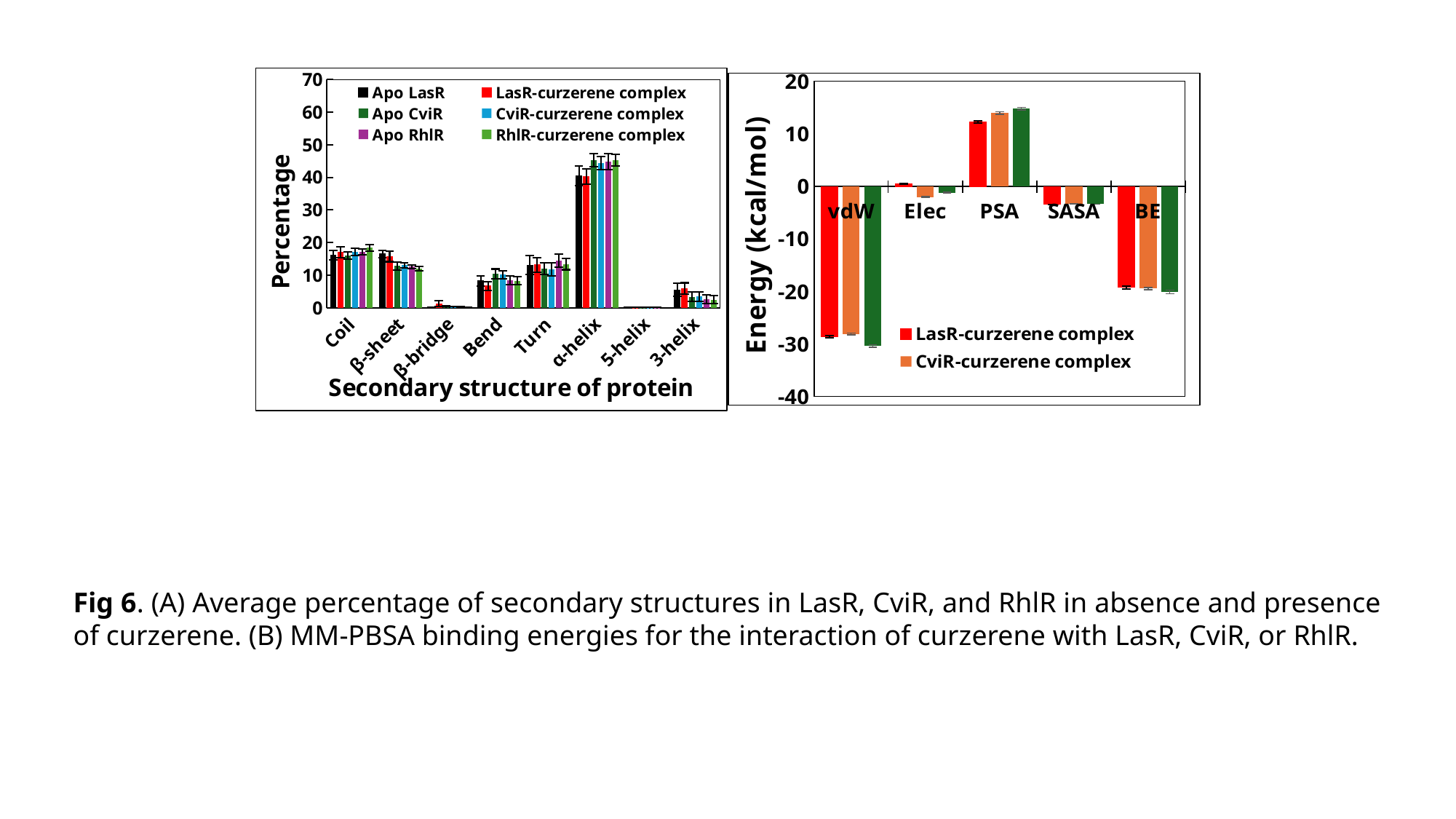

### Chart
| Category | Apo LasR | LasR-curzerene complex | Apo CviR | CviR-curzerene complex | Apo RhlR | RhlR-curzerene complex |
|---|---|---|---|---|---|---|
| Coil | 16.117512693780263 | 17.06075962721566 | 15.930359744784111 | 17.12924347401436 | 17.114087555932798 | 18.387193930508506 |
| β-sheet | 16.52749204760275 | 15.736729954622572 | 12.83424589982402 | 13.031576951541961 | 12.584951889290192 | 12.062815652637108 |
| β-bridge | 0.044530971106392224 | 1.2266258404760764 | 0.1795656804894001 | 0.15303712165933347 | 0.06148147963768242 | 0.0038996350107571845 |
| Bend | 8.232218586957455 | 6.715834747160998 | 10.40787858364582 | 10.103556977847457 | 8.391101862615029 | 8.317506623156696 |
| Turn | 13.088057235178733 | 13.154178980154889 | 12.017437150020196 | 11.708096627686416 | 14.436199896950072 | 13.432666163862661 |
| α-helix | 40.50637725215694 | 40.19404708015452 | 45.26162496584349 | 44.4155790355873 | 44.75868311814815 | 45.35474647809028 |
| 5-helix | 0.0018401227729914142 | 0.0024534970306552186 | 0.00318661367328128 | 0.00318661367328128 | 0.003733693095405815 | 0.00041485478837842394 |
| 3-helix | 5.481971090444487 | 5.909370273184626 | 3.365701361719688 | 3.4557231979898844 | 2.649760504330669 | 2.440756661945619 |
### Chart
| Category | LasR-curzerene complex | CviR-curzerene complex | RhlR-curzerene complex |
|---|---|---|---|
| vdW | -28.618307839388144 | -28.090583173996176 | -30.367351816443595 |
| Elec | 0.5336998087954111 | -1.9961759082217974 | -1.1708891013384322 |
| PSA | 12.284177820267686 | 13.972753346080307 | 14.836281070745699 |
| SASA | -3.4385755258126194 | -3.335325047801147 | -3.3580305927342256 |
| BE | -19.230879541108987 | -19.44455066921606 | -20.05282026768642 |Fig 6. (A) Average percentage of secondary structures in LasR, CviR, and RhlR in absence and presence of curzerene. (B) MM-PBSA binding energies for the interaction of curzerene with LasR, CviR, or RhlR.
